# Supplementary material for: Disparities between sustainability of country-level seafood production and consumption
Source: PLoS One. 2024 Dec 2;19(12):e0313823. doi: 10.1371/journal.pone.0313823 (PMC11611205; doi:10.1371/journal.pone.0313823)
Supplement: S1 Table — The GTA data is recorded in product weight, while the FAO data is recorded in live weight. To combine information from both datasets it was necessary to convert the product weights to live weights in the GTA data. For shark fins we used an estimate based on previously published studies investigating the fin to body weight ratio of blue sharks (Prionace glauca), one of the most commonly caught species in shark fisheries (1–3). For livers/roes/milts, fish heads/fins/maws, and caviar/caviar substitutes we used the ratios for cod livers/roes suggested by the FAO’s Measures, Stowage Rates and Yields of Fishery Products publication (4). Additionally, we categorized each GTA product into the corresponding FAO ISSCAAP group. Below is a list of all products in the GTA data, the live weight conversion factors we used, the motivation behind the conversion factor, and the associated FAO product group. (PDF) [file pone.0313823.s001.pdf]

Table S1. Live weight conversion factors and FAO Product Groups. The GTA data is recorded in product weight, while the FAO data is recorded in live weight. To combine information from both datasets it was necessary to convert the product weights to live weights in the GTA data. For shark fins we used an estimate based on previously published studies investigating the fin to body weight ratio of blue sharks (Prionace glauca), one of the most commonly caught species in shark fisheries (1-3). For livers/roes/milts, fish heads/fins/maws, and caviar/caviar substitutes we used the ratios for cod livers/roes suggested by the FAO's Measures, Stowage Rates and Yields of Fishery Products publication (4). Additionally, we categorized each GTA product into the corresponding FAO ISSCAAP group. Below is a list of all products in the GTA data, the live weight conversion factors we used, the motivation behind the conversion factor, and the associated FAO product group.

HS6 Code	Product Type	CF	CF Decision	FAO Group
030100	Live Fish	1	Reported in live weight	Marine fish nei
030191	Fish; live, trout (*Salmo trutta*, *Salmo gairdneri*, *Salmo clarki*, *Salmo aguabonita*, *Salmo gilae*)	1	Reported in live weight	Freshwater & diadromous fish
030192	Fish; live, eels (*Anguilla spp*.)	1	Reported in live weight	Freshwater & diadromous fish
030193	Fish; live, carp	1	Reported in live weight	Freshwater & diadromous fish
030194	Fish; live, bluefin tunas (*Thunnus thynnus*)	1	Reported in live weight	Pelagic fish
030195	Fish; live, southern bluefin tunas (*Thunnus maccoyii*)	1	Reported in live weight	Pelagic fish
030199	Fish; live, n.e.s. in heading no. 0301	1	Reported in live weight	Marine fish nei
030200	Fresh or Chilled Fish	1.286	FAO Assumed; average of all frozen fish CFs	Marine fish nei
030211	Fish; trout (*Salmo trutta, Salmo gairdneri*, *Salmo clarki*, *Salmo aguabonita*, *Salmo gilae*), fresh or chilled	1.4	FAO Assumed; frozen trout gutted/head on	Freshwater & diadromous fish

	(excluding fillets, livers, roes and other fish meat of heading no. 0304)			
030212	Fish; Pacific salmon (*Oncorhynchus spp.*), Atlantic salmon (*Salmo salar*), Danube salmon (*Hucho hucho*), fresh or chilled (excluding fillets, livers, roes and other fish meat of heading no. 0304)	1.143	FAO Assumed; average of frozen salmon whole gutted/head on, dressed gutted/head on, dressed gutted/head off	Freshwater & diadromous fish
030213	Fish; fresh or chilled, Pacific salmon (*Oncorhynchus nerka, Oncorhynchus gorbuscha, Oncorhynchus keta, Oncorhynchus tschawytscha, Oncorhynchus kisutch, Oncorhynchus masou, Oncorhynchus rhodurus*), not fillets, livers, roes, other fish meat of heading 0304	1.143	FAO Assumed; average of frozen salmon whole gutted/head on, dressed gutted/head on, dressed gutted/head off	Freshwater & diadromous fish
030214	Fish; fresh or chilled, Atlantic salmon (*Salmo salar*) and Danube salmon (*Hucho hucho*), excluding fillets, livers, roes, and other fish meat of heading 0304	1.143	FAO Assumed; average of frozen salmon whole gutted/head on, dressed gutted/head on, dressed gutted/head off	Freshwater & diadromous fish
030219	Fish; salmonidae, fresh or chilled, n.e.s. in item no. 0302.1 (excluding fillets, livers, roes and other fish meat of heading no. 0304)	1.143	FAO Assumed; average of frozen salmon whole gutted/head on, dressed gutted/head on, dressed gutted/head off	Freshwater & diadromous fish
030221	Fish; halibut (*Reinhardtius hippoglossoides, Hippoglossus hippoglossus, Hippoglossus stenolepis*), fresh or chilled (excluding fillets, livers, roes and other fish meat of heading no. 0304)	1.25	FAO Assumed; average of frozen flatfish dressed gutted/head on, gutted/head off	Demersal fish
030222	Fish; plaice (*Pleuronectes platessa*), fresh or chilled (excluding fillets, livers, roes and other fish meat of heading no. 0304)	1.25	FAO Assumed; average of frozen flatfish dressed gutted/head on, gutted/head off	Demersal fish
030223	Fish; sole (*Solea spp.*), fresh or chilled (excluding fillets, livers, roes and other fish meat of heading no. 0304)	1.25	FAO Assumed; average of frozen flatfish dressed gutted/head on, gutted/head off	Demersal fish

030224	Fish; fresh or chilled, turbots (*Psetta maxima*, Scophthalmidae), excluding fillets, livers, roes, and other fish meat of heading 0304	1.25	FAO Assumed; average of frozen flatfish dressed gutted/head on, gutted/head off	Demersal fish
030229	Fish; flat fish, fresh or chilled, n.e.s. in item no. 0302.2 (excluding fillets, livers, roes and other fish meat of heading no. 0304)	1.25	FAO Assumed; average of frozen flatfish dressed gutted/head on, gutted/head off	Demersal fish
030231	Fish; albacore or longfinned tunas (*Thunnus alalunga*), fresh or chilled (excluding fillets, livers, roes and other fish meat of heading no. 0304)	1.26	FAO Assumed; average of frozen other tunas gutted/head on, gutted/head off	Pelagic fish
030232	Fish; yellowfin tunas (*Thunnus albacares*), fresh or chilled (excluding fillets, livers, roes and other fish meat of heading no. 0304)	1.1	FAO Assumed; frozen yellowfin gutted/head on	Pelagic fish
030233	Fish; skipjack or stripe-bellied bonito, fresh or chilled (excluding fillets, livers, roes and other fish meat of heading no. 0304)	1.1	FAO Assumed; frozen skipjack gutted/head on	Pelagic fish
030234	Fish; bigeye tunas (*Thunnus obesus*), fresh or chilled (excluding fillets, livers, roes and other fish meat of heading no. 0304)	1.26	FAO Assumed; average of frozen other tunas gutted/head on, gutted/head off	Pelagic fish
030235	Fish; bluefin tunas (*Thunnus thynnus*), fresh or chilled (excluding fillets, livers, roes and other fish meat of heading no. 0304)	1.26	FAO Assumed; average of frozen other tunas gutted/head on, gutted/head off	Pelagic fish
030236	Fish; southern bluefin tunas (*Thunnus maccoyii*), fresh or chilled (excluding fillets, livers, roes and other fish meat of heading no. 0304)	1.26	FAO Assumed; average of frozen other tunas gutted/head on, gutted/head off	Pelagic fish
030239	Fish; tuna, fresh or chilled, n.e.s. in item no. 0302.3 (excluding fillets, livers, roes and other fish meat of heading no. 0304)	1.26	FAO Assumed; average of frozen other tunas gutted/head on, gutted/head off	Pelagic fish

030240	Fish; herrings (*Clupea harengus*, *Clupea pallasii*), fresh or chilled (excluding fillets, livers, roes and other fish meat of heading no. 0304)	1.26	FAO Assumed; average of frozen whole herring gutted/head on, gutted/head off	Pelagic fish
030241	Fish; fresh or chilled, herrings (*Clupea harengus*, *Clupea pallasii*), excluding fillets, livers, roes, and other fish meat of heading 0304	1.26	FAO Assumed; average of frozen whole herring gutted/head on, gutted/head off	Pelagic fish
030242	Fish; fresh or chilled, anchovies (*Engraulis spp.*), excluding fillets, livers, roes, and other fish meat of heading 0304	1	FAO Assumed; frozen anchovy gutted/head on	Pelagic fish
030243	Fish; fresh or chilled, sardines (*Sardina pilchardus*, *Sardinops spp.*), sardinella (*Sardinella spp.*), brisling or sprats (*Sprattus sprattus*), excluding fillets, livers, roes, and other fish meat of heading 0304	1.215	FAO Assumed; average of frozen sardine gutted/head on, gutted/head off	Pelagic fish
030244	Fish; fresh or chilled, mackerel (*Scomber scombrus*, *Scomber australasicus*, *Scomber japonicus*), excluding fillets, livers, roes, and other fish meat of heading 0304	1.285	FAO Assumed; average frozen mackerel gutted/head on, gutted/head off	Pelagic fish
030245	Fish; fresh or chilled, jack and horse mackerel (*Trachurus spp.*), excluding fillets, livers, roes, and other fish meat of heading 0304	1.37	FAO Assumed; average frozen jack and horse mackerel gutted/head on, gutted/head off	Pelagic fish
030246	Fish; fresh or chilled, cobia (*Rachycentron canadum*), excluding fillets, livers, roes, and other fish meat of heading 0304	1.29	FAO Assumed; frozen bonito gutted/head off	Pelagic fish
030247	Fish; fresh or chilled, swordfish (*Xiphias gladius*), excluding fillets, livers, roes, and other fish meat of heading 0304	1.31	FAO Assumed; frozen swordfish gutted/head off	Pelagic fish
030249	Fish; fresh or chilled, n.e.c. in item no. 0302.4, excluding fillets, fish meat of 0304, and edible fish offal of subheadings 0302.91 to 0302.99	1.286	FAO Assumed; average of all frozen fish CFs	Marine fish nei
030250	Fish; cod (*Gadus morhua*, *Gadus ogac*, *Gadus macrocephalus*), fresh or chilled (excluding fillets, livers, roes and other fish meat of heading no. 0304)	1.445	FAO Assumed; average frozen cod gutted/head on, gutted/head off	Demersal fish

030251	Fish; fresh or chilled, cod (*Gadus morhua*, *Gadus ogac*, *Gadus macrocephalus*), excluding fillets, livers, roes, and other fish meat of heading 0304	1.445	FAO Assumed; average frozen cod gutted/head on, gutted/head off	Demersal fish
030252	Fish; fresh or chilled, haddock (*Melanogrammus aeglefinus*), excluding fillets, livers, roes, and other fish meat of heading 0304	1.375	FAO Assumed; average frozen haddock gutted/head on, gutted/head off	Demersal fish
030253	Fish; fresh or chilled, coalfish (*Pollachius virens*), excluding fillets, livers, roes, and other fish meat of heading 0304	1.3	FAO Assumed; average frozen saithe gutted/head on, gutted/head off	Demersal fish
030254	Fish; fresh or chilled, hake (*Merluccius spp.*, *Urophycis spp.*), excluding fillets, livers, roes, and other fish meat of heading 0304	1.237	FAO Assumed; average frozen hake dressed gutted/head on, dressed gutted/head off, whole gutted/head on	Demersal fish
030255	Fish; fresh or chilled, Alaska pollack (*Theragra chalcogramma*), excluding fillets, livers, roes, and other fish meat of heading 0304	2.17	FAO Assumed; frozen AK pollack dressed gutted/head off	Demersal fish
030256	Fish; fresh or chilled, blue whitings (*Micromesistius poutassou*, *Micromesistius australis*), excluding fillets, livers, roes, and other fish meat of heading 0304	1.412	FAO Assumed; average of all frozen gadidae CFs	Demersal fish
030259	Fish; fresh or chilled, n.e.c. in item no. 0302.5, excluding fillets, livers, roes, and other fish meat of heading 0304	1.286	FAO Assumed; average of all frozen fish CFs	Marine fish nei
030260	Fish; fresh or chilled, n.e.c. in item no. 0302.5, excluding fillets, livers, roes, and other fish meat of heading 0304	1.286	FAO Assumed; average of all frozen fish CFs	Marine fish nei
030261	Fish; sardines (*Sardina pilchardus*, *Sardinops spp.*), sardinella (*Sardinella spp.*), brisling or sprats (*Sprattus sprattus*), fresh or chilled (excluding fillets, livers, roes and other fish meat of heading no. 0304)	1.215	FAO Assumed; average of frozen sardine gutted/head on, gutted/head off	Pelagic fish
030262	Fish; haddock (*Melanogrammus aeglefinus*), fresh or chilled (excluding fillets, livers, roes and other fish meat of heading no. 0304)	1.375	FAO Assumed; average of frozen haddock gutted/head on, gutted/head off	Pelagic fish

030263	Fish; coalfish (*Pollachius virens*), fresh or chilled (excluding fillets, livers, roes and other fish meat of heading no. 0304)	1.3	FAO Assumed; average frozen saithe gutted/head on, gutted/head off	Demersal fish
030264	Fish; mackerel (*Scomber scombrus*, *Scomber australasicus*, *Scomber japonicus*), fresh of chilled (excluding fillets, livers, roes and other fish meat of heading no. 0304)	1.285	FAO Assumed; frozen mackerel gutted/head on, gutted/head off	Pelagic fish
030265	Fish; dogfish and other sharks, fresh or chilled (excluding fillets, livers, roes and other fish meat of heading no. 0304)	1.55	FAO Assumed; average of frozen shark gutted/head on, gutted/head off	Demersal fish
030266	Fish; eels (*Anguilla spp.*), fresh or chilled (excluding fillets, livers, roes and other fish meat of heading no. 0304)	1.11	FAO Assumed; frozen eel gutted/head on	Freshwater & diadromous fish
030267	Fish; swordfish (*Xiphias gladius*), fresh or chilled (excluding fillets, livers, roes and other fish meat of heading no. 0304)	1.31	FAO Assumed; frozen swordfish gutted/head off	Pelagic fish
030268	Fish; toothfish (*Dissostichus spp.*), fresh or chilled (excluding fillets, livers, roes and other fish meat of heading no. 0304)	1.412	FAO Assumed; average of all frozen gadidae CFs	Demersal fish
030269	Fish; fresh or chilled, n.e.s. in heading no. 0302 (excluding fillets, livers, roes and other fish meat of heading no. 0304)	1.286	FAO Assumed; average of all frozen fish CFs	Marine fish nei
030270	Fish; livers and roes, fresh or chilled	20	FAO Waterman 2001 ratio for cod	Marine fish nei
030271	Fish; fresh or chilled, tilapias (*Oreochromis spp.*), excluding fillets, livers, roes, and other fish meat of heading 0304	1.163	FAO Assumed; average of all frozen freshwater/anadromous fish	Freshwater & diadromous fish
030272	Fish; fresh or chilled, catfish (*Pangasius spp.*, *Silurus spp.*, *Clarias spp.*, *Ictalurus spp.*), excluding fillets, livers, roes, and other fish meat of heading 0304	1.163	FAO Assumed; average of all frozen freshwater/anadromous fish	Freshwater & diadromous fish
030273	Fish; fresh or chilled, carp (*Cyprinus carpio*, *Carassius carassius*, *Ctenopharyngodon idellus*, *Hypophthalmichthys spp.*, *Cirrhinus spp.*,	1.163	FAO Assumed; average of all frozen freshwater/anadromous fish	Freshwater & diadromous fish

	Mylopharyngodon piceus), excluding fillets, livers, roes, and other fish meat of heading 0304			
030274	Fish; fresh or chilled, eels (*Anguilla spp.*), excluding fillets, livers, roes, and other fish meat of heading 0304	1.11	FAO Assumed; frozen eel gutted/head on	Freshwater & diadromous fish
030279	Fish; fresh or chilled, Nile perch (*Lates niloticus*) and snakeheads (*Channa spp.*), excluding fillets, livers, roes, and other fish meat of heading 0304	1.163	FAO Assumed; average of all frozen freshwater/anadromous fish	Freshwater & diadromous fish
030281	Fish; fresh or chilled, dogfish and other sharks, excluding fillets, livers, roes, and other fish meat of heading 0304	1.55	FAO Assumed; average of frozen shark gutted/head on, gutted/head off	Demersal fish
030282	Fish; fresh or chilled, rays and skates (Rajidae), excluding fillets, livers, roes, and other fish meat of heading 0304	1.55	FAO Assumed; average of frozen shark gutted/head on, gutted/head off	Demersal fish
030283	Fish; fresh or chilled, toothfish (*Dissostichus spp.*), excluding fillets, livers, roes, and other fish meat of heading 0304	1.412	FAO Assumed; average of all frozen gadidae CFs	Demersal fish
030284	Fish; fresh or chilled, seabass (*Dicentrarchus spp.*), excluding fillets, livers, roes, and other fish meat of heading 0304	1.455	FAO Assumed; average of frozen sea bass gutted/head on, gutted/head off	Marine fish nei
030285	Fish; fresh or chilled, seabream (Sparidae), excluding fillets, livers, roes, and other fish meat of heading 0304	1.285	FAO Assumed; average of sea bream gutted/head on, gutted/head off	Marine fish nei
030289	Fish; fresh or chilled, n.e.c. in heading 0302, excluding fillets, livers, roes, and other fish meat of heading 0304	1.286	FAO Assumed; average of all frozen fish CFs	Marine fish nei
030290	Fish; fresh or chilled, livers and roes	20	FAO Waterman 2001 ratio for cod	Marine fish nei
030291	Fish; fresh or chilled, livers, roes and milt	20	FAO Waterman 2001 ratio for cod	Marine fish nei
030292	Fish; fresh or chilled, shark fins	15.6	Fin/body ratio from prev. studies	Demersal fish
030299	Fish; fresh or chilled, fish fins (other than shark fins), heads, tails, maws and other edible fish offal	20	FAO Waterman 2001 ratio for cod	Marine fish nei

030300	Fish; frozen	1.286	FAO Assumed; average of all frozen fish CFs	Marine fish nei
030310	Fish; Pacific salmon, (*Oncorhynchus spp.*), frozen (excluding fillets, livers, roes and other fish meat of heading no. 0304)	1.143	FAO Direct; average of whole gutted/head on, dressed gutted/head on, dressed gutted/head off	Freshwater & diadromous fish
030311	Fish; Pacific salmon, sockeye salmon, (red salmon), (*Oncorhynchus nerka*) frozen, (excluding fillets, livers, roes and other fish meat of heading no. 0304)	1.143	FAO Direct; average of whole gutted/head on, dressed gutted/head on, dressed gutted/head off	Freshwater & diadromous fish
030312	Fish; frozen, Pacific salmon (*Oncorhynchus gorbuscha/keta/tschawytscha/ kisutch/masou/rhodurus*) other than sockeye salmon (*Oncorhynchus nerka*), excluding fillets, livers, roes, and other fish meat of heading 0304	1.143	FAO Direct; average of whole gutted/head on, dressed gutted/head on, dressed gutted/head off	Freshwater & diadromous fish
030313	Fish; frozen, Atlantic salmon (*Salmo salar*) and Danube salmon (*Hucho hucho*), excluding fillets, livers, roes, and other fish meat of heading 0304	1.143	FAO Direct; average of whole gutted/head on, dressed gutted/head on, dressed gutted/head off	Freshwater & diadromous fish
030314	Fish; frozen, trout (*Salmo trutta, Oncorhynchus mykiss, Oncorhynchus clarki, Oncorhynchus aguabonita, Oncorhynchus gilae, Oncorhynchus apache* and *Oncorhynchus chrysogaster*), excluding fillets, livers, roes, and other fish meat of heading 0304	1.4	FAO Direct; gutted/head on	Freshwater & diadromous fish
030319	Fish; Pacific salmon, other than sockeye salmon, (*Oncorhynchus gorbuscha/keta/tschawytscha/kisutch/masou/rhodurus*), frozen, (excluding fillets, livers, roes and other fish meat of heading no. 0304)	1.143	FAO Direct; average of whole gutted/head on, dressed gutted/head on, dressed gutted/head off	Freshwater & diadromous fish
030321	Fish; trout (*Salmo trutta, Salmo gairdneri, Salmo clarki, Salmo aguabonita, Salmo gilae*), frozen (excluding fillets, livers, roes and other fish meat of heading no. 0304)	1.4	FAO Direct; gutted/head on	Freshwater & diadromous fish
030322	Fish; Atlantic salmon (*Salmo salar*) and Danube salmon (*Hucho hucho*), frozen (excluding fillets, livers, roes and other fish meat of heading no. 0304)	1.143	FAO Direct; average of whole gutted/head on,	Freshwater & diadromous fish

			dressed gutted/head on, dressed gutted/head off	
030323	Fish; frozen, tilapias (*Oreochromis spp.*), excluding fillets, livers, roes, and other fish meat of heading 0304	1.163	FAO Assumed; average of all frozen freshwater/anadromous fish	Freshwater & diadromous fish
030324	Fish; frozen, catfish (*Pangasius spp.*, *Silurus spp.*, *Clarias spp.*, *Ictalurus spp.*), excluding fillets, livers, roes, and other fish meat of heading 0304	1.163	FAO Assumed; average of all frozen freshwater/anadromous fish	Freshwater & diadromous fish
030325	Fish; frozen, carp (*Cyprinus carpio*, *Carassius carassius*, *Ctenopharyngodon idellus*, *Hypophthalmichthys spp.*, *Cirrhinus spp.*, *Mylopharyngodon piceus*), excluding fillets, livers, roes, and other fish meat of heading 0304	1.163	FAO Assumed; average of all frozen freshwater/anadromous fish	Freshwater & diadromous fish
030326	Fish; frozen, eels (*Anguilla spp.*), excluding fillets, livers, roes, and other fish meat of heading 0304	1.11	FAO Direct; gutted/head on	Freshwater & diadromous fish
030329	Fish; salmonidae, frozen, n.e.s. in item no. 0302.1 and 0302.2 (excluding fillets, livers, roes and other fish meat of heading no. 0304)	1.143	FAO Direct; average of whole gutted/head on, dressed gutted/head on, dressed gutted/head off	Freshwater & diadromous fish
030331	Fish; halibut (*Reinhardtius hippoglossoides*, *Hippoglossus hippoglossus*, *Hippoglossus stenolepis*), frozen (excluding fillets, livers, roes and other fish meat of heading no. 0304)	1.25	FAO Direct; average of flatfish dressed gutted/head on; dressed gutted/head off	Demersal fish
030332	Fish; plaice (*Pleuronectes platessa*), frozen (excluding fillets, livers, roes and other fish meat of heading no. 0304)	1.25	FAO Direct; average of flatfish dressed gutted/head on; dressed gutted/head off	Demersal fish
030333	Fish; sole (*Solea spp.*), frozen (excluding fillets, livers, roes and other fish meat of heading no. 0304)	1.25	FAO Direct; average of flatfish dressed gutted/head on; dressed gutted/head off	Demersal fish

030334	Fish; frozen, turbots (*Psetta maxima*, Scophthalmidae), excluding fillets, livers, roes, and other fish meat of heading 0304	1.25	FAO Direct; average of flatfish dressed gutted/head on; dressed gutted/head off	Demersal fish
030339	Fish; flat fish, frozen, n.e.s. in item no. 0303.3 (excluding fillets, livers, roes and other fish meat of heading no. 0304)	1.25	FAO Direct; average of flatfish dressed gutted/head on; dressed gutted/head off	Demersal fish
030341	Fish; albacore or longfinned tunas (*Thunnus alalunga*), frozen (excluding fillets, livers, roes and other fish meat of heading no. 0304)	1.26	FAO Direct; average of Other tunas gutted/head on, gutted/head off	Pelagic fish
030342	Fish; yellowfin tunas (*Thunnus albacares*), frozen (excluding fillets, livers, roes and other fish meat of heading no. 0304)	1.1	FAO Direct; yellowfin gutted/head on	Pelagic fish
030343	Fish; skipjack or stripe-bellied bonito, frozen (excluding fillets, livers, roes and other fish meat of heading no. 0304)	1.1	FAO Direct; skipjack gutted/head on	Pelagic fish
030344	Fish; bigeye tunas (*Thunnus obesus*), frozen, (excluding fillets, livers, roes and other fish meat of heading no. 0304)	1.26	FAO Direct; average of Other tunas gutted/head on, gutted/head off	Pelagic fish
030345	Fish; bluefin tuna (*Thunnus thynnus*), frozen, (excluding fillets, livers, roes and other fish meat of heading no. 0304)	1.26	FAO Direct; average of Other tunas gutted/head on, gutted/head off	Pelagic fish
030346	Fish; southern bluefin tunas (*Thunnus maccoyii*), frozen, n.e.c. in item no. 0303.4 (excluding fillets, livers, roes and other fish meat of heading no. 0304)	1.26	FAO Direct; average of Other tunas gutted/head on, gutted/head off	Pelagic fish
030349	Fish; tuna, frozen, n.e.s. in item no. 0303.4 (excluding fillets, livers, roes and other fish meat of heading no. 0304)	1.26	FAO Direct; average of Other tunas gutted/head on, gutted/head off	Pelagic fish
030350	Fish; herrings (*Clupea harengus, Clupea pallasii*), frozen (excluding fillets, livers, roes and other fish meat of heading no. 0304)	1.26	FAO Direct; average of herring gutted/head on, gutted/head off	Pelagic fish
030351	Fish; herrings (*Clupea harengus, Clupea pallasii*), frozen (excluding fillets, livers, roes and other fish meat of heading no. 0304)	1.26	FAO Direct; average of herring gutted/head on, gutted/head off	Pelagic fish

030352	Fish; cod (*Gadus morhua, Gadus ogac, Gadus macrocephalus*), frozen (excluding fillets, livers, roes and other fish meat of heading no. 0304)	1.445	FAO Direct; average of cod gutted/head on, cod gutted/head off	Demersal fish
030353	Fish; frozen, sardines (*Sardina pilchardus, Sardinops spp.*), sardinella (*Sardinella spp.*), brisling or sprats (*Sprattus sprattus*), excluding fillets, livers, roes, and other fish meat of heading 0304	1.143	FAO Direct; average of sardine, sprat gutted/head on, gutted/head off	Pelagic fish
030354	Fish; frozen, mackerel (*Scomber scombrus, Scomber australasicus*, *Scomber japonicus*), excluding fillets, livers, roes, and other fish meat of heading 0304	1.285	FAO Direct; average of mackerel gutted/head on, gutted/head off	Pelagic fish
030355	Fish; frozen, jack and horse mackerel (*Trachurus spp.*), excluding fillets, livers, roes, and other fish meat of heading 0304	1.37	FAO Direct; average of jack and horse mackerel gutted/head on, gutted/head off	Pelagic fish
030356	Fish; frozen, cobia (*Rachycentron canadum*), excluding fillets, livers, roes, and other fish meat of heading 0304	1.29	FAO Assumed; frozen bonito gutted/head off	Marine fish nei
030357	Fish; frozen, swordfish (*Xiphias gladius*), excluding fillets, livers, roes, and other fish meat of heading 0304	1.31	FAO Direct; swordfish gutted/head off	Pelagic fish
030359	Fish; frozen, n.e.c. in item no. 0303.5, excluding fillets, fish meat of 0304, and edible fish offal of subheadings 0303.91 to 0303.99	1.286	FAO Assumed; average of all frozen fish CFs	Marine fish nei
030360	Fish; cod (*Gadus morhua, Gadus ogac, Gadus macrocephalus*), frozen (excluding fillets, livers, roes and other fish meat of heading no. 0304)	1.445	FAO Direct; average of cod gutted/head on, cod gutted/head off	Demersal fish
030361	Fish; swordfish (*Xiphias gladius*), frozen (excluding fillets, livers, roes and other fish meat of heading no. 0304)	1.31	FAO Direct; swordfish gutted/head off	Pelagic fish
030362	Fish; toothfish (*Dissostichus spp.*), frozen (excluding fillets, livers, roes and other fish meat of heading no. 0304)	1.412	FAO Assumed; average of all frozen gadidae CFs	Demersal fish
030363	Fish; frozen, cod (*Gadus morhua, Gadus ogac, Gadus macrocephalus*), excluding fillets, livers, roes, and other fish meat of heading 0304	1.445	FAO Direct; average of cod gutted/head on, cod gutted/head off	Demersal fish
030364	Fish; frozen, haddock (*Melanogrammus aeglefinus*), excluding fillets, livers, roes, and other fish meat of heading 0304	1.375	FAO Direct; average of haddock gutted/head on	Demersal fish

			and haddock gutted/head off	
030365	Fish; frozen, coalfish (*Pollachius virens*), excluding fillets, livers, roes, and other fish meat of heading 0304	1.3	FAO Assumed; average frozen saithe gutted/head on, gutted/head off	Demersal fish
030366	Fish; frozen, hake (*Merluccius spp.*, *Urophycis spp.*), excluding fillets, livers, roes, and other fish meat of heading 0304	1.237	FAO Direct; average of hake dressed gutted/head on, dressed gutted/head off, whole gutted/head on	Demersal fish
030367	Fish; frozen, Alaska pollack (*Theraga chalcogramma*), excluding fillets, livers, roes, and other fish meat of heading 0304	2.17	FAO Direct; Alaska pollack dressed gutted/head off	Demersal fish
030368	Fish; frozen, blue whitings (*Micromesistius poutassou*, *Micromesistius australis*), excluding fillets, livers, roes, and other fish meat of heading 0304	1.412	FAO Assumed; average of all frozen gadidae CFs	Demersal fish
030369	Fish; frozen, of Bregmacerotidae, Euclichthyidae, Gadidae, Macrouridae, Melanonidae, Merlucciidae, Moridae, Muraenolepididae, other than cod, haddock, coalfish, hake, Alaska pollack, blue whitings, excluding fillets, livers, roes, other fish meat of 0304	1.412	FAO Assumed; average of all frozen gadidae CFs	Demersal fish
030371	Fish; sardines (*Sardina pilchardus*, *Sardinops spp.*), sardinella (*Sardinella spp.*), brisling or sprats (*Sprattus sprattus*), frozen (excluding fillets, livers, roes and other fish meat of heading no. 0304)	1.143	FAO Direct; average of sardine, sprat gutted/head on, gutted/head off	Pelagic fish
030372	Fish; haddock (*Melanogrammus aeglefinus*), frozen (excluding fillets, livers, roes and other fish meat of heading no. 0304)	1.375	FAO Direct; average of haddock gutted/head on and haddock gutted/head off	Demersal fish
030373	Fish; coalfish (*Pollachius virens*), frozen (excluding fillets, livers, roes and other fish meat of heading no. 0304)	1.3	FAO Assumed; average frozen saithe gutted/head on, gutted/head off	Demersal fish
030374	Fish; mackerel (*Scomber scombrus*, *Scomber australasicus*, *Scomber japonicus*), frozen (excluding fillets, livers, roes and other fish meat of heading no. 0304)	1.285	FAO Direct; average of mackerel gutted/head on, gutted/head off	Pelagic fish

030375	Fish; dogfish and other sharks, frozen (excluding fillets, livers, roes and other fish meat of heading no. 0304)	1.55	FAO Direct; average of shark gutted/head on, gutted head/off	Demersal fish
030376	Fish; eels (*Anguilla spp.*), frozen (excluding fillets, livers, roes and other fish meat of heading no. 0304)	1.11	FAO Direct; gutted/head on	Freshwater & diadromous fish
030377	Fish; sea bass (*Dicentrarchus labrax, Dicentrarchus punctatus*), frozen, (excluding fillets, livers, roes and other fish meat of heading no. 0304)	1.455	FAO Direct; average of sea bass gutted/head on, gutted/headoff	Marine fish nei
030378	Fish; hake (*Merluccius spp., Urophycis spp.*), frozen (excluding fillets, livers, roes and other fish meat of heading no. 0304)	1.237	FAO Direct; average of hake dressed gutted/head on, dressed gutted/head off, whole gutted/head on	Demersal fish
030379	Fish; frozen, n.e.s. in heading no. 0303 (excluding fillets, livers, roes and other fish meat of heading no. 0304)	1.286	FAO Assumed; average of all frozen fish CFs	Marine fish nei
030380	Fish; livers and roes, frozen	20	FAO Waterman 2001 ratio for cod	Marine fish nei
030381	Fish; frozen, dogfish and other sharks, excluding fillets, livers, roes, and other fish meat of heading 0304	1.55	FAO Direct; average of shark gutted/head on, gutted head/off	Demersal fish
030382	Fish; frozen, rays and skates (Rajidae), excluding fillets, livers, roes, and other fish meat of heading 0304	1.55	FAO Direct; average of shark gutted/head on, gutted head/off	Demersal fish
030383	Fish; frozen, toothfish (*Dissostichus spp.*), excluding fillets, livers, roes, and other fish meat of heading 0304	1.412	FAO Assumed; average of all frozen gadidae CFs	Demersal fish
030384	Fish; frozen, seabass (*Dicentrarchus spp.*), excluding fillets, livers, roes, and other fish meat of heading 0304	1.455	FAO Direct; average of sea bass gutted/head on, gutted/headoff	Marine fish nei
030389	Fish; frozen, n.e.c. in heading 0303, excluding fillets, livers, roes, and other fish meat of heading 0304	1.286	FAO Assumed; average of all frozen fish CFs	Marine fish nei
030390	Fish; frozen, livers and roes	20	FAO Waterman 2001 ratio for cod	Marine fish nei
030391	Fish; frozen, livers, roes and milt	20	FAO Waterman 2001 ratio for cod	Marine fish nei

030392	Fish; frozen, shark fins	15.6	Fin/body ratio from prev. studies	Demersal fish
030399	Fish; frozen, fish fins (other than shark fins), heads, tails, maws and other edible fish offal	20	FAO Waterman 2001 ratio for cod	Marine fish nei
030400	Fish; Fillets and Other Meat, fresh or chilled	2.712	FAO Assumed; average of all fillet CFs	Marine fish nei
030410	Fish; fillets and other fish meat, fresh or chilled (whether or not minced)	2.712	FAO Assumed; average of all fillet CFs	Marine fish nei
030411	Fish; swordfish (*Xiphias gladius*), fillets and other fish meat (whether or not minced), fresh or chilled	1.73	FAO Assumed; average of raw, skin off tuna CFs	Pelagic fish
030412	Fish; toothfish (*Dissostichus spp.*), fillets and other fish meat (whether or not minced), fresh or chilled	3.04	FAO Assumed; average of raw, skin off gadidae CFs	Demersal fish
030419	Fish; fillets and other fish meat (whether or not minced), fresh or chilled, other than swordfish (*Xiphias gladius*) and toothfish (*Dissostichus spp.*)	2.712	FAO Assumed; average of all fillet CFs	Demersal fish
030420	Fish; fillets, frozen	2.712	FAO Assumed; average of all fillet CFs	Marine fish nei
030421	Fish; swordfish (*Xiphias gladius*), fillets, frozen	1.73	FAO Assumed; average of raw, skin off tuna CFs	Pelagic fish
030422	Fish; toothfish (*Dissostichus spp.*), fillets, frozen	3.043	FAO Assumed; average of raw, skin off gadidae CFs	Demersal fish
030429	Fish; fillets, frozen, other than swordfish (*Xiphias gladius*) and toothfish (*Dissostichus spp.*)	2.712	FAO Assumed; average of all fillet CFs	Pelagic fish
030431	Fish fillets; fresh or chilled, tilapias (*Oreochromis spp.*)	3.09	FAO Assumed; average of catfish fillet raw CF and skin off CF	Freshwater & diadromous fish
030432	Fish fillets; fresh or chilled, catfish (*Pangasius spp., Silurus spp., Clarias spp., Ictalurus spp.*)	3.09	FAO Direct; average of catfish fillet raw CF and skin off CF	Freshwater & diadromous fish
030433	Fish fillets; fresh or chilled, Nile perch (*Lates niloticus*)	3.09	FAO Assumed; average of catfish fillet raw CF and skin off CF	Freshwater & diadromous fish
030439	Fish fillets; fresh or chilled, carp (*Cyprinus carpio, Carassius carassius, Ctenopharyngodon idellus, Hypophthalmichthys spp., Cirrhinus spp.,*	3.09	FAO Assumed; average of catfish fillet raw CF and skin off CF	Freshwater & diadromous fish

	Mylopharyngodon piceus), eels (*Anguilla spp.*), and snakeheads (*Channa spp.*)			
030441	Fish fillets; fresh or chilled, salmon, Pacific (*Oncorhynchus nerka, Oncorhynchus gorbuscha, Oncorhynchus keta, Oncorhynchus tschawytscha, Oncorhynchus kisutch, Oncorhynchus masou* and *Oncorhynchus rhodurus*), Atlantic (*Salmo salar*), Danube (*Hucho hucho*)	2	FAO Direct; salmon fillets skin off, raw CF	Freshwater & diadromous fish
030442	Fish fillets; fresh or chilled, trout (*Salmo trutta, Oncorhynchus mykiss, Oncorhynchus clarki, Oncorhynchus aguabonita, Oncorhynchus gilae, Oncorhynchus apache* and *Oncorhynchus chrysogaster*)	2	FAO Assumed; salmon fillets skin off, raw CFs	Freshwater & diadromous fish
030443	Fish fillets; fresh or chilled, flat fish (Pleuronectidae, Bothidae, Cynoglossidae, Soleidae, Scophthalmidae and Citharidae)	2.545	FAO Direct; average of flatfish fillets raw CF, skin off CF	Demersal fish
030444	Fish fillets; fresh or chilled, of the families Bregmacerotidae, Euclichthyidae, Gadidae, Macrouridae, Melanonidae, Merlucciidae, Moridae, and Muraenolepididae	3.043	FAO Assumed; average of raw, skin off gadidae CFs	Demersal fish
030445	Fish fillets; fresh or chilled, swordfish (*Xiphias gladius*)	1.73	FAO Assumed; average of raw, skin off tuna CFs	Pelagic fish
030446	Fish fillets; fresh or chilled, toothfish (*Dissostichus spp.*)	3.043	FAO Assumed; average of raw, skin off gadidae CFs	Demersal fish
030447	Fish fillets; fresh or chilled, dogfish and other sharks	1.73	FAO Assumed; average of raw, skin off tuna CFs	Demersal fish
030448	Fish fillets; fresh or chilled, rays and skates (Rajidae)	1.73	FAO Assumed; average of raw, skin off tuna CFs	Demersal fish
030449	Fish fillets; fresh or chilled, other than fish of heading 0304.4	2.712	FAO Assumed; average of all fillet CFs	Marine fish nei
030451	Fish meat, excluding fillets, whether or not minced; fresh or chilled, tilapias, catfish, carp, eels, Nile perch, and snakeheads	3.09	FAO Assumed; average of catfish fillet raw CF and skin off CF	Freshwater & diadromous fish
030452	Fish meat, excluding fillets, whether or not minced; fresh or chilled, salmonidae	2	FAO Direct; salmon fillets skin off CF	Freshwater & diadromous fish

030453	Fish meat, excluding fillets, whether or not minced; fresh or chilled, of the families Bregmacerotidae, Euclichthyidae, Gadidae, Macrouridae, Melanonidae, Merlucciidae, Moridae, and Muraenolepididae	3.043	FAO Assumed; average of raw, skin off gadidae CFs	Demersal fish
030454	Fish meat, excluding fillets, whether or not minced; fresh or chilled, swordfish (*Xiphias gladius*)	1.73	FAO Assumed; average of raw, skin off tuna CFs	Pelagic fish
030455	Fish meat, excluding fillets, whether or not minced; fresh or chilled, toothfish (*Dissostichus spp.*)	3.043	FAO Assumed; average of raw, skin off gadidae CFs	Demersal fish
030456	Fish meat; excluding fillets, whether or not minced; fresh or chilled, dogfish and other sharks	1.73	FAO Assumed; average of raw, skin off tuna CFs	Demersal fish
030457	Fish meat; excluding fillets, whether or not minced; fresh or chilled, rays and skates (Rajidae)	1.73	FAO Assumed; average of raw, skin off tuna CFs	Demersal fish
030459	Fish meat; excluding fillets, whether or not minced; fresh or chilled, of fish n.e.c. in item no. 0304.5	2.712	FAO Assumed; average of all fillet CFs	Marine fish nei
030461	Fish fillets; frozen, tilapias (*Oreochromis spp.*)	3.09	FAO Assumed; average of catfish fillet raw CF and skin off CF	Freshwater & diadromous fish
030462	Fish fillets; frozen, catfish (*Pangasius spp., Silurus spp., Clarias spp., Ictalurus spp.*)	3.09	FAO Direct; average of catfish fillet raw CF and skin off CF	Freshwater & diadromous fish
030463	Fish fillets; frozen, Nile Perch (*Lates niloticus*)	3.09	FAO Assumed; average of catfish fillet raw CF and skin off CF	Freshwater & diadromous fish
030469	Fish fillets; frozen, carp (*Cyprinus carpio, Carassius carassius, Ctenopharyngodon idellus, Hypophthalmichthys spp., Cirrhinus spp., Mylopharyngodon piceus*), eels (*Anguilla spp.*), and snakeheads (*Channa spp.*)	3.09	FAO Assumed; average of catfish fillet raw CF and skin off CF	Freshwater & diadromous fish
030471	Fish fillets; frozen, cod (*Gadus morhua, Gadus ogac, Gadus macrocephalus*)	2.97	FAO Direct; average of cod fillet raw CF and skin off CF options	Demersal fish
030472	Fish fillets; frozen, haddock (*Melanogrammus aeglefinus*)	2.83	FAO Direct; average of haddock fillet faw CF and skin off CF options	Demersal fish

030473	Fish fillets; frozen, coalfish (*Pollachius virens*)	2.335	FAO Assumed; average of saithe fillet raw CF and skin CFs	Demersal fish
030474	Fish fillets; frozen, hake (*Merluccius spp., Urophycis spp.*)	2.777	FAO Direct; average of hake fillet raw CF and skin off CF options	Demersal fish
030475	Fish fillets; frozen, Alaska pollack (*Theraga chalcogramma*)	3.78	FAO Direct; AK pollack fillet skin off	Demersal fish
030479	Fish fillets; frozen, of the families Bregmacerotidae, Euclichthyidae, Gadidae, Macrouridae, Melanonidae, Merlucciidae, Moridae and Muraenolepididae other than cod, haddock, coalfish, hake, and Alaska pollack	3.043	FAO Assumed; average of raw, skin off gadidae CFs	Demersal fish
030481	Fish fillets; frozen, salmon, Pacific (*Oncorhynchus nerka, Oncorhynchus gorbuscha, Oncorhynchus keta, Oncorhynchus tschawytscha, Oncorhynchus kisutch, Oncorhynchus masou, Oncorhynchus rhodurus*), Atlantic (*Salmo salar*), and Danube (*Hucho hucho*)	2	FAO Direct; salmon fillets skin off CF	Freshwater & diadromous fish
030482	Fish fillets; frozen, trout (*Salmo trutta, Oncorhynchus mykiss, Oncorhynchus clarki, Oncorhynchus aguabonita, Oncorhynchus gilae, Oncorhynchus apache* and *Oncorhynchus chrysogaster*)	2	FAO Assumed; salmon fillets skin off, raw CFs	Freshwater & diadromous fish
030483	Fish fillets; frozen, flat fish (Pleuronectidae, Bothidae, Cynoglossidae, Soleidae, Scophthalmidae and Citharidae)	2.545	FAO Direct; average of flatfish fillets raw CF, skin off CF	Demersal fish
030484	Fish fillets; frozen, swordfish (*Xiphias gladius*)	1.73	FAO Assumed; average of raw, skin off tuna CFs	Pelagic fish
030485	Fish fillets; frozen, toothfish (*Dissostichus spp.*)	3.043	FAO Assumed; average of raw, skin off gadidae CFs	Demersal fish
030486	Fish fillets; frozen, herrings (*Clupea harengus, Clupea pallasii*)	1.895	FAO Direct; average of herring fillets raw CF and skin off CF	Pelagic fish
030487	Fish fillets; frozen, tunas (of the genus Thunnus), skipjack or stripe-bellied bonito (*Euthynnus (Katsuwonus) pelamis*)	1.73	FAO Direct; average of tuna fillets raw CF and skin off CF	Pelagic fish
030488	Fish fillets; frozen, dogfish, other sharks, rays and skates (Rajidae)	1.73	FAO Assumed; average of raw, skin off tuna CFs	Demersal fish

030489	Fish fillets; frozen, of fish n.e.c. in heading 0304.8	2.712	FAO Assumed; average of all fillet CFs	Marine fish nei
030490	Fish; fish meat n.e.s. in heading no. 0304 (whether or not minced), fresh, chilled or frozen	2.712	FAO Assumed; average of all fillet CFs	Marine fish nei
030491	Fish; swordfish (*Xiphias gladius*), frozen, other than fillets	1.73	FAO Assumed; average of raw, skin off tuna CFs	Pelagic fish
030492	Fish; toothfish (*Dissostichus spp.*), frozen, other than fillets	3.043	FAO Assumed; average of raw, skin off gadidae CFs	Demersal fish
030493	Fish meat, excluding fillets, whether or not minced; frozen, tilapias, catfish, carp, eels, Nile perch, and snakeheads	3.09	FAO Assumed; average of catfish fillet raw CF and skin off CF	Freshwater & diadromous fish
030494	Fish meat, excluding fillets, whether or not minced; frozen, Alaska Pollack (*Theraga chalcogramma*)	3.78	FAO Assumed; average of AK pollack fillet CFs	Demersal fish
030495	Fish meat, excluding fillets, whether or not minced; frozen, of the families Bregmacerotidae, Euclichthyidae, Gadidae, Macrouridae, Melanonidae, Merlucciidae, Moridae and Muraenolepididae, other than Alaska Pollack (*Theraga chalcogramma*)	3.043	FAO Assumed; average of raw, skin off gadidae CFs	Demersal fish
030496	Fish meat, excluding fillets, whether or not minced; frozen, dogfish and other sharks	1.73	FAO Assumed; average of raw, skin off tuna CFs	Demersal fish
030497	Fish meat, excluding fillets, whether or not minced; frozen, rays and skates (Rajidae)	1.73	FAO Assumed; average of raw, skin off tuna CFs	Demersal fish
030499	Fish; fish meat (whether or not minced) other than fillets, frozen, excluding swordfish (*Xiphias gladius*) and toothfish (*Dissostichus spp.*)	2.712	FAO Assumed; average of all fillet CFs	Marine fish nei
030500	Fish Dried, Salted, or in Brine	2.294	FAO Assumed; average of dried/salted/brined deduced CFs	Marine fish nei
030510	Fish meal; fit for human consumption	1	Base assumption due to lack of information	Marine fish nei
030520	Fish; livers and roes, dried, smoked (whether or not cooked before or during the smoking process), salted or in brine	20	FAO Waterman 2001 ratio for cod	Marine fish nei
030530	Fish; fillets, dried, salted or in brine, but not smoked	2.294	FAO Assumed; average of dried/salted/brined deduced CFs	Marine fish nei

030531	Fish fillets; dried, salted or in brine, but not smoked, tilapias, catfish, carp, eels, Nile perch, and snakeheads	2.48	FAO Direct; average of deduced CFs of dry gutted tilapia, freshwater fillet, salted/wet/brined freshwater fish	Freshwater & diadromous fish
030532	Fish fillets; dried, salted or in brine, but not smoked, of the families Bregmacerotidae, Euclichthyidae, Gadidae, Macrouridae, Melanonidae, Merlucciidae, Moridae and Muraenolepididae	2.722	FAO Assumed; average of dried/salted/brined gadidae and other demersal deduced CFs	Demersal fish
030539	Fish fillets; dried, salted or in brine, but not smoked, n.e.c. in item no. 0305.3	2.294	FAO Assumed; average of dried/salted/brined deduced CFs	Marine fish nei
030541	Fish; Pacific salmon (*Oncorhynchus spp*.), Atlantic salmon (*Salmo salar*) and Danube salmon (*Hucho hucho*), including fillets, smoked (whether or not cooked before or during the smoking process)	1.92	FAO Direct; deduced CF for smoked salmon	Freshwater & diadromous fish
030542	Fish; herrings (*Clupea harengus*, *Clupea pallasii*), including fillets; smoked (whether or not cooked before or during the smoking process)	1.49	FAO Direct; deduced CF for smoked herring	Pelagic fish
030543	Fish; smoked, whether or not cooked before or during smoking, trout (*Salmo trutta, Oncorhynchus mykiss/clarki/aguabonita/gilae/apache/chrysogaster*), includes fillets, but excludes edible fish offal	1.92	FAO Assumed, deduced CF for smoked salmon	Freshwater & diadromous fish
030544	Fish; smoked, whether or not cooked before or during smoking, tilapias, catfish, carp, eels, Nile perch, and snakeheads, includes fillets, but excludes edible fish offal	1.43	FAO Assumed; deduced CF for smoked eel	Freshwater & diadromous fish
030549	Fish; smoked (whether or not cooked before or during the smoking process), n.e.s. in item no. 0305.4 (including fillets)	1.683	FAO Assumed; average of all smoked fish deduced CFs	Marine fish nei
030551	Fish; cod (*Gadus morhua, Gadus ogac, Gadus macrocephalus*), dried (whether or not salted but not smoked)	2.955	FAO Direct; average of deduced CFs of dried gutted cod and filleted cod	Demersal fish
030552	Fish; dried, whether or not salted but not smoked, tilapias, catfish, carp, eels, Nile perch, and snakeheads	2.97	FAO Direct; average of deduced CFS of dried	Freshwater & diadromous fish

			gutted tilapia and other freshwater fish fillets	
030553	Fish; dried, whether or not salted but not smoked, fish of the families Bregmacerotidae, Euclichthyidae, Gadidae, Macrouridae, Melanonidae, Merlucciidae, Moridae and Muraenolepididae, other than cod	3.337	FAO Assumed; average of deduced CFs of dried gadidae	Demersal fish
030554	Dried herrings, anchovies, sardines, sardinella, brisling or sprats, mackerel (incl Indian, jack, or horse), seerfishes, jacks, crevalles, cobia, silver pomfrets, Pacific saury, scads, capelin, swordfish, Kawakawa, bonitos, marlins, sailfishes, spearfish	3.037	FAO Assumed; average of deduced CFs of dried pilchard, tuna, shark	Pelagic fish
030559	Fish; dried (whether or not salted but not smoked), n.e.s. in item no. 0305.51	3.217	FAO Assumed; average of all deduced CFs of dried fish	Marine fish nei
030561	Fish; herrings (*Clupea harengas*, *Clupea pallasii*), salted or in brine but not dried or smoked	1.39	FAO Direct; recommended CF for salted/wet/brined herring	Pelagic fish
030562	Fish; cod (*Gadus morhua, Gadus ogac, Gadus macrocephalus*), salted or in brine but not dried or smoked	2	FAO Direct; recommended CF for salted/wet/brined cod	Demersal fish
030563	Fish; anchovies (*Engraulis spp.*), salted or in brine but not dried or smoked	1.33	FAO Direct; recommended CF for salted/wet/brined anchovies	Pelagic fish
030564	Fish; salted or in brine, not dried or smoked, other than edible fish offal, tilapias, catfish, carp, eels, Nile perch, and snakeheads	1.5	FAO Direct; recommend CF for salted/wet/brined freshwater fish	Freshwater & diadromous fish
030569	Fish; salted or in brine, but not dried or smoked, n.e.s. in item no. 0305.6	1.464	FAO Assumed; average of all recommended CFs for salted/wet/brined fish	Marine fish nei
030571	Fish; edible offal, shark fins	15.6	Fin/body ratio from prev. studies	Demersal fish
030572	Fish; edible offal, fish heads, tails and maws	20	FAO Waterman 2001 ratio for cod	Marine fish nei
030579	Fish; edible offal, other than shark fins, fish heads, tails and maws	20	FAO Waterman 2001 ratio for cod	Marine fish nei

030600	Crustaceans	3.287	FAO Assumed; average of all crustacean indicative CFs	Crustaceans
030611	Crustaceans; rock lobsters and other sea crawfish (*Palinurus spp., Panulirus spp., Jasus spp.*), frozen (whether in shell or not, whether or not cooked by steaming or by boiling in water)	3.245	FAO Direct; average of lobsters tails (shell on), meat indicative CFs	Crustaceans
030612	Crustaceans; lobsters (*Homarus spp.*), frozen (whether in shell or not, whether or not cooked by steaming or by boiling in water)	3.245	FAO Direct; average of lobsters tails (shell on), meat indicative CFs	Crustaceans
030613	Crustaceans; shrimps and prawns, frozen (whether in shell or not, whether or not cooked by steaming or by boiling in water)	2.41	FAO Direct; average of shrimps/prawns tails (shell on), tails (peeled), meat indicative CFs	Crustaceans
030614	Crustaceans; crabs, frozen (whether in shell or not, whether or not cooked by steaming or by boiling in water)	6	FAO Direct; crab meat indicative CF	Crustaceans
030615	Crustaceans; frozen, Norway lobsters (*Nephrops norvegicus*), in shell or not, smoked, cooked or not before or during smoking; in shell, cooked by steaming or by boiling in water	3.245	FAO Direct; average of lobsters tails (shell on), meat indicative CFs	Crustaceans
030616	Crustaceans; frozen, cold-water shrimps and prawns (*Pandalus spp., Crangon crangon*), in shell or not, smoked, cooked or not before or during smoking; in shell, cooked by steaming or by boiling in water	2.41	FAO Direct; average of shrimps/prawns tails (shell on), tails (peeled), meat indicative CFs	Crustaceans
030617	Crustaceans; frozen, shrimps and prawns, excluding cold-water varieties, in shell or not, smoked, cooked or not before or during smoking; in shell, cooked by steaming or by boiling in water	2.41	FAO Direct; average of shrimps/prawns tails (shell on), tails (peeled), meat indicative CFs	Crustaceans
030619	Crustaceans; frozen, n.e.s. in item no. 0306.1 (whether in shell or not, whether or not cooked by steaming or by boiling in water)	3.287	FAO Assumed; average of all crustacean indicative CFs	Crustaceans
030620	Crustaceans	3.287	FAO Assumed; average of all crustacean indicative CFs	Crustaceans

030621	Crustaceans; rock lobster and other sea crawfish (*Palinurus spp.*, *Panulirus spp.*, *Jasus spp.*), not frozen, (whether in shell or not, whether or not cooked by steaming or by boiling in water)	3.245	FAO Direct; average of lobsters tails (shell on), meat indicative CFs	Crustaceans
030622	Crustaceans; lobsters (*Homarus spp.*), not frozen, (whether in shell or not, whether or not cooked by steaming or by boiling in water)	3.245	FAO Direct; average of lobsters tails (shell on), meat indicative CFs	Crustaceans
030623	Crustaceans; shrimps and prawns, not frozen, (whether in shell or not, whether or not cooked by steaming or by boiling in water)	2.41	FAO Direct; average of shrimps/prawns tails (shell on), tails (peeled), meat indicative CFs	Crustaceans
030624	Crustaceans; crabs, not frozen, (whether in shell or not, whether or not cooked by steaming or by boiling in water)	6	FAO Direct; crab meat indicative CF	Crustaceans
030625	Crustaceans; not frozen, Norway lobsters (*Nephrops norvegicus*), in shell or not, smoked, cooked or not before or during smoking; in shell, cooked by steaming or by boiling in water; edible flours, meals, and pellets	3.245	FAO Direct; average of lobsters tails (shell on), meat indicative CFs	Crustaceans
030626	Crustaceans; not frozen, cold-water shrimps and prawns (*Pandalus spp.*, *Crangon crangon*), in shell or not, smoked, cooked or not before or during smoking; in shell, cooked by steaming or by boiling in water; edible flour, meals, and pellets	2.41	FAO Direct; average of shrimps/prawns tails (shell on), tails (peeled), meat indicative CFs	Crustaceans
030627	Crustaceans; not frozen, shrimps and prawns excluding cold-water varieties, in shell or not, smoked, cooked or not before or during smoking; in shell, cooked by steaming or by boiling in water; edible flour, meals, and pellets	2.41	FAO Direct; average of shrimps/prawns tails (shell on), tails (peeled), meat indicative CFs	Crustaceans
030629	Crustaceans; not frozen, n.e.s. in heading no. 0306, (whether in shell or not, whether or not cooked by steaming or by boiling in water)	3.287	FAO Assumed; average of all crustacean indicative CFs	Crustaceans
030631	Crustaceans; live, fresh or chilled, rock lobsters and other sea crawfish (*Palinurus spp.*, *Panulirus spp.*, *Jasus spp.*), in shell or not	3.245	FAO Direct; average of lobsters tails (shell on), meat indicative CFs	Crustaceans

030632	Crustaceans; live, fresh or chilled, lobsters (*Homarus spp.*), whether in shell or not	3.245	FAO Direct; average of lobsters tails (shell on), meat indicative CFs	Crustaceans
030633	Crustaceans; live, fresh or chilled, crabs, whether in shell or not	6	FAO Direct; crab meat indicative CF	Crustaceans
030634	Crustaceans; live, fresh or chilled, Norway lobsters (*Nephrops norvegicus*), in shell or not	3.245	FAO Direct; average of lobsters tails (shell on), meat indicative CFs	Crustaceans
030635	Crustaceans; live, fresh or chilled, cold-water shrimps and prawns (*Pandalus spp., Crangon crangon*), in shell or not	2.41	FAO Direct; average of shrimps/prawns tails (shell on), tails (peeled), meat indicative CFs	Crustaceans
030636	Crustaceans; live, fresh or chilled, shrimps and prawns excluding cold-water varieties, in shell or not	2.41	FAO Direct; average of shrimps/prawns tails (shell on), tails (peeled), meat indicative CFs	Crustaceans
030639	Crustaceans; live, fresh or chilled, n.e.c. in item no. 0306.3, in shell or not, including edible flours, meals, and pellets of crustaceans	3.287	FAO Assumed; average of all crustacean indicative CFs	Crustaceans
030691	Crustaceans; rock lobsters and other sea crawfish (*Palinurus spp., Panulirus spp., Jasus spp.*), smoked, cooked or not, whether in shell or not, whether or not cooked before or during smoking	3.245	FAO Direct; average of lobsters tails (shell on), meat indicative CFs	Crustaceans
030692	Crustaceans; lobsters (*Homarus spp.*), smoked, cooked or not, whether in shell or not, whether or not cooked before or during smoking	3.245	FAO Direct; average of lobsters tails (shell on), meat indicative CFs	Crustaceans
030693	Crustaceans; crabs, smoked, cooked or not, whether in shell or not, whether or not cooked before or during smoking	6	FAO Direct; crab meat indicative CF	Crustaceans
030694	Crustaceans; Norway lobsters (*Nephrops norvegicus*), smoked, cooked or not, whether in shell or not, whether or not cooked before or during smoking	3.245	FAO Direct; average of lobsters tails (shell on), meat indicative CFs	Crustaceans
030695	Crustaceans; shrimps and prawns, smoked, cooked or not, whether in shell or not, whether or not cooked before or during smoking	2.41	FAO Direct; average of shrimps/prawns tails (shell on), tails (peeled), meat indicative CFs	Crustaceans

030699	Crustaceans; smoked, whole, cooked or not, n.e.c. in item no. 0306.9, in shell or not, including edible flours, meals, and pellets of crustaceans	3.287	FAO Assumed; average of all crustacean indicative CFs	Crustaceans
030700	Molluscs	4.573	FAO Assumed; average of all mollusc indicative CFs	Molluscs excl. cephalopods
030710	Molluscs; oysters, live, fresh, chilled, frozen, dried, salted or in brine (whether in shell or not)	10	FAO Direct; average of oyster indicative CFs	Molluscs excl. cephalopods
030711	Molluscs; oysters, whether in shell or not, live, fresh or chilled	10	FAO Direct; not dried oyster indicative CFs	Molluscs excl. cephalopods
030712	Molluscs; oysters, whether in shell or not, frozen	10	FAO Direct; not dried oyster indicative CFs	Molluscs excl. cephalopods
030719	Molluscs; oysters, whether in shell or not, frozen, dried, salted or in brine, smoked, cooked or not before or during the smoking process	10	FAO Direct; average of oyster indicative CFs	Molluscs excl. cephalopods
030721	Molluscs; scallops (including queen scallops of the genera *Pecten*, *Chlamys* or *Placopecten*), live, fresh or chilled (whether in shell or not)	9.1	FAO Direct; not dried scallop indicative CF	Molluscs excl. cephalopods
030722	Molluscs; scallops, whether in shell or not, including queen scallops of the genera *Pecten*, *Chlamys* or *Placopecten*, frozen	9.1	FAO Direct; not dried scallop indicative CF	Molluscs excl. cephalopods
030729	Molluscs; scallops (including queen scallops of the genera *Pecten*, *Chlamys* or *Placopecten*), frozen, dried, salted or in brine (whether in shell or not)	9.85	FAO Direct; average of scallop indicative CFs	Molluscs excl. cephalopods
030731	Molluscs; mussels (*Mytilus spp.*, *Perna spp.*), live, fresh or chilled (whether in shell or not)	6	FAO Direct; mussel indicative CF	Molluscs excl. cephalopods
030732	Molluscs; mussels (*Mytilus spp.*, *Perna spp.*), whether in shell or not, frozen	6	FAO Direct; mussel indicative CF	Molluscs excl. cephalopods
030739	Molluscs; mussels (*Mytilus spp.*, *Perna spp.*), frozen, dried, salted or in brine (whether in shell or not)	6	FAO Direct; mussel indicative CF	Molluscs excl. cephalopods
030741	Molluscs; cuttle fish and squid, live, fresh or chilled (whether in shell or not)	1.773	FAO Direct; average of squid whole/raw indicative CFs	Cephalopods
030742	Molluscs; cuttle fish and squid, whether in shell or not, live, fresh or chilled	1.773	FAO Direct; average of squid whole/raw indicative CFs	Cephalopods

030743	Molluscs; cuttle fish and squid, whether in shell or not, includes flours, meals, and pellets of molluscs, fit for human consumption, frozen	1.615	FAO Direct; average of squid whole/raw, cuttlefish frozen/gutted indicative CFs	Cephalopods
030749	Molluscs; cuttle fish and squid, frozen, dried, salted or in brine (whether in shell or not)	1.871	FAO Direct; average of all squid and cuttelfish indicative CFs	Cephalopods
030751	Molluscs; octopus (*Octopus spp.*), live, fresh or chilled	1.3	FAO Assumed; frozen octopus indicative CF	Cephalopods
030752	Molluscs; octopus (*Octopus spp.*), frozen	1.3	FAO Direct; frozen octopus indicative CF	Cephalopods
030759	Molluscs; octopus (octopus spp.), frozen, dried, salted or in brine	2.65	FAO Direct; average of all octopus indicative CFs	Cephalopods
030760	Molluscs; snails (other than sea snails), live, fresh, chilled, frozen, dried, salted or in brine (whether in shell or not)	2.44	FAO Direct; abalone indicative CF	Molluscs excl. cephalopods
030771	Molluscs; clams, cockles and ark shells (families Arcidae, Arcticidae, Cardiidae, Donacidae, Hiatellidae, Mactridae, Mesodesmatidae, Myidae, Semelidae, Solecurtidae, Solenidae, Tridacnidae and Veneridae), whether in shell or not, live, fresh or chilled	8.3	FAO Direct; average of clam indicative CFs	Molluscs excl. cephalopods
030772	Molluscs; clams, cockles, ark shells (Arcidae, Arcticidae, Cardiidae, Donacidae, Hiatellidae, Mactridae, Mesodesmatidae, Myidae, Semelidae, Solecurtidae, Solenidae, Tridacnidae and Veneridae), whether in shell or not, frozen	8.3	FAO Direct; average of clam indicative CFs	Molluscs excl. cephalopods
030779	Molluscs; clams, cockles, ark shells (Arcidae, Arcticidae, Cardiidae, Donacidae, Hiatellidae, Mactridae, Mesodesmatidae, Myidae, Semelidae, Solecurtidae, Solenidae, Tridacnidae and Veneridae), whether in shell or not, other than live, fresh or chilled	8.3	FAO Direct; average of clam indicative CFs	Molluscs excl. cephalopods
030781	Molluscs; abalone (*Haliotis spp.*), whether in shell or not, live, fresh or chilled	2.44	FAO Direct; abalone indicative CF	Molluscs excl. cephalopods
030782	Molluscs; stromboid conchs (*Strombus spp.*), whether in shell or not, live, fresh or chilled	2.44	FAO Assumed; abalone indicative CF	Molluscs excl. cephalopods

030783	Molluscs; abalone (*Haliotis spp*.), whether in shell or not, frozen	2.44	FAO Direct; abalone indicative CF	Molluscs excl. cephalopods
030784	Molluscs; stromboid conchs (*Strombus spp*.), whether in shell or not, frozen	2.44	FAO Assumed; abalone indicative CF	Molluscs excl. cephalopods
030787	Molluscs; abalone (*Haliotis spp*.), whether in shell or not, dried, salted, in brine, or smoked, cooked or not before or during the smoking process	2.44	FAO Direct; abalone indicative CF	Molluscs excl. cephalopods
030788	Molluscs; stromboid conchs (*Strombus spp*.), whether in shell or not, includes flours, meals, and pellets of molluscs, fit for human consumption, dried, salted, in brine, or smoked, cooked or not before or during the smoking process	2.44	FAO Assumed; abalone indicative CF	Molluscs excl. cephalopods
030789	Molluscs; abalone (*Haliotis spp*.), whether in shell or not, frozen, dried, salted, in brine, or smoked, cooked or not before or during the smoking process	2.44	FAO Direct; abalone indicative CF	Molluscs excl. cephalopods
030791	Molluscs and other aquatic invertebrates; live, fresh or chilled (whether in shell or not), n.e.s. in heading no. 0307	4.264	FAO Assumed; average of all crustaceans and molluscs indicative CFs	Molluscs excl. cephalopods
030792	Molluscs; n.e.c. in heading 0307, whether in shell or not, includes flours, meals, and pellets of molluscs, fit for human consumption, frozen	4.264	FAO Assumed; average of all crustaceans and molluscs indicative CFs	Molluscs excl. cephalopods
030799	Molluscs and other aquatic invertebrates; frozen, dried, salted or in brine (whether in shell or not), n.e.s. in heading no. 0307	4.264	FAO Assumed; average of all crustaceans and molluscs indicative CFs	Molluscs excl. cephalopods
030811	Aquatic invertebrates; sea cucumbers (*Stichopus japonicus*, Holothurioidea), live, fresh or chilled	4.264	FAO Assumed; average of all crustaceans and molluscs indicative CFs	Aquatic animals nei
030812	Aquatic invertebrates; sea cucumbers (*Stichopus japonicus*, Holothuroidea), frozen	4.264	FAO Assumed; average of all crustaceans and molluscs indicative CFs	Aquatic animals nei
030819	Aquatic invertebrates; sea cucumbers (*Stichopus japonicus*, Holothurioidea), frozen, dried, salted or in brine, smoked, whether or not cooked before or during the smoking process	4.264	FAO Assumed; average of all crustaceans and molluscs indicative CFs	Aquatic animals nei

030821	Aquatic invertebrates; sea urchins (*Strongylocentrotus spp.*, *Paracentrotus lividus*, *Loxechinus albus*, *Echichinus esculentus*), live, fresh or chilled	4.264	FAO Assumed; average of all crustaceans and molluscs indicative CFs	Aquatic animals nei
030822	Aquatic invertebrates; sea urchins (*Strongylocentrotus spp.*, *Paracentrotus lividus*, *Loxechinus albus*, *Echinus esculentus*), frozen	4.264	FAO Assumed; average of all crustaceans and molluscs indicative CFs	Aquatic animals nei
030829	Aquatic invertebrates; sea urchins (*Strongylocentrotus spp.*, *Paracentrotus lividus*, *Loxechinus albus*, *Echichinus esculentus*), frozen, dried, salted or in brine, smoked, whether or not cooked before or during smoking process	4.264	FAO Assumed; average of all crustaceans and molluscs indicative CFs	Aquatic animals nei
030830	Aquatic invertebrates; jellyfish (*Rhopilema spp.*), live, fresh, chilled, frozen, dried, salted or in brine, smoked, whether or not cooked before or during smoking	4.264	FAO Assumed; average of all crustaceans and molluscs indicative CFs	Aquatic animals nei
030890	Aquatic invertebrates; other than crustaceans, molluscs, sea urchins, sea cucumbers and jellyfish, live, fresh, chilled, frozen, dried, salted or in brine, smoked, cooked or not before or during smoking, includes edible flours, meals, and pellets	4.264	FAO Assumed; average of all crustaceans and molluscs indicative CFs	Aquatic animals nei
030917	No Description	1	Base assumption due to lack of information	Marine fish nei
031095	Young Fish (Fingerling)	1	Base assumption due to lack of information	Marine fish nei
039269	Other	1	Base assumption due to lack of information	Marine fish nei
039290	No Description	1	Base assumption due to lack of information	Marine fish nei
160400	Fish preparations; caviar and caviar substitutes	20	FAO Waterman 2001 ratio for cod	Marine fish nei
160411	Fish preparations; salmon, prepared or preserved, whole or in pieces (but not minced)	1.56	FAO Direct; average of prepared/preserved/canned salmon steak bone in, salmon/smoked derived CFs	Freshwater & diadromous fish
160412	Fish preparations; herrings, prepared or preserved, whole or in pieces (but not minced)	1.08	FAO Direct; average of prepared/preserved/canned	Pelagic fish

			filleted herring, semi-preserved/marinated gutted/fillet herring derived CFs	
160413	Fish preparations; sardines, sardinella and brisling or sprats, prepared or preserved, whole or in pieces (but not minced)	1.07	FAO Assumed; prepared/preserved/canned gutted sardine derived CF	Pelagic fish
160414	Fish preparations; tunas, skipjack and Atlantic bonito (sarda spp.), prepared or preserved, whole or in pieces (but not minced)	1.44	FAO Direct; prepared/preserved/canned albacore, bonito, other tuna flesh/boneless derived CF	Pelagic fish
160415	Fish preparations; mackerel, prepared or preserved, whole or in pieces (but not minced)	1.46	FAO Direct; prepared/preserved/canned mackerel derived CF	Pelagic fish
160416	Fish preparations; anchovies, prepared or preserved, whole or in pieces (but not minced)	1.63	FAO Direct; salted anchovy fillets in containers derived CF	Pelagic fish
160417	Fish preparations; eels, prepared or preserved, whole or in pieces (but not minced)	2.03	FAO Direct; prepared/preserved/canned eel fillet derived CF	Freshwater & diadromous fish
160418	Fish preparations; shark fins, prepared or preserved, whole or in pieces (but not minced)	15.6	Fin/body ratio from prev. studies	Demersal fish
160419	Fish preparations; fish prepared or preserved, whole or in pieces (but not minced), n.e.s. in heading no. 1604	1.631875	FAO Assumed; average of all prepared/preserved/canned preparations in containers derived CFs	Marine fish nei
160420	Fish preparations; fish minced or in forms n.e.s. in heading no. 1604, prepared or preserved	1.938	FAO Assumed; average of fish balls, cakes, ham, sausages, paste, fermented CFs	Marine fish nei
160430	Fish preparations; caviar and caviar substitutes	20	FAO Waterman 2001 ratio for cod	Marine fish nei
160431	Fish preparations; caviar	20	FAO Waterman 2001 ratio for cod	Marine fish nei

160432	Fish preparations; caviar substitutes, prepared from fish eggs	20	FAO Waterman 2001 ratio for cod	Marine fish nei
160500	Crustacean preparations; prepared or preserved crustaceans (excluding crab, shrimps, prawns and lobster)	3.5	FAO Direct; average of other crustaceans canned and fermented crustaceans in containers derived CFs	Crustaceans
160510	Crustacean preparations; crab, prepared or preserved	4.5	FAO Direct; canned crab and preparations of crab in containers	Crustaceans
160520	Crustacean preparations; shrimps and prawns, prepared or preserved	2.053	FAO Direct; average of shrimp/prawn canned and preparations in containers derived CFs	Crustaceans
160521	Crustacean preparations; shrimps and prawns, prepared or preserved, not in airtight containers	2.03	FAO Direct; average of shrimp/prawn preparations in containers derived CFs	Crustaceans
160529	Crustacean preparations; shrimps and prawns, prepared or preserved, in airtight containers	2.053	FAO Direct; average of shrimp/prawn canned and preparations in containers derived CFs	Crustaceans
160530	Crustacean preparations; lobster, prepared or preserved	2.9	FAO Direct; canned lobster derived CF	Crustaceans
160540	Crustacean preparations; prepared or preserved crustaceans (excluding crab, shrimps, prawns and lobster)	3.5	FAO Direct; average of other crustaceans canned and fermented crustaceans in containers derived CFs	Crustaceans
160551	Mollusc preparations; oysters, prepared or preserved	7.5	FAO Direct, canned oysters derived CF	Molluscs excl. cephalopods
160552	Mollusc preparations; scallops, including queen scallops, prepared or preserved	6.8	FAO Direct; canned scallops derived CF	Molluscs excl. cephalopods
160553	Mollusc preparations; mussels, prepared or preserved	4.5	FAO Direct; canned mussels derived CF	Molluscs excl. cephalopods
160554	Mollusc preparations; cuttle fish and squid, prepared or preserved	1.09	FAO Direct; canned squid, etc. derived CF	Cephalopods
160555	Mollusc preparations; octopus, prepared or preserved	1.09	FAO Direct; canned squid, etc. derived CF	Cephalopods

160556	Mollusc preparations; clams, cockles and arkshells, prepared or preserved	4.5	FAO Direct; canned clams derived CF	Molluscs excl. cephalopods
160557	Mollusc preparations; abalone, prepared or preserved	1.83	FAO Direct; canned abalone derived CF	Molluscs excl. cephalopods
160558	Mollusc preparations; snails, other than sea snails, prepared or preserved	1.83	FAO Assumed; canned abalone derived CF	Molluscs excl. cephalopods
160559	Mollusc preparations; n.e.c. in item no. 1605.5, prepared or preserved	3.321	FAO Assumed; average of all canned/preparations in containers derived CFs	Molluscs excl. cephalopods
160561	Aquatic invertebrates; sea cucumbers, prepared or preserved	3.321	FAO Assumed; average of all canned/preparations in containers derived CFs	Aquatic animals nei
160562	Aquatic invertebrates; sea urchins, prepared or preserved	3.321	FAO Assumed; average of all canned/preparations in containers derived CFs	Aquatic animals nei
160563	Aquatic invertebrates; jellyfish, prepared or preserved	3.321176471	FAO Assumed; average of all canned/preparations in containers derived CFs	Aquatic animals nei
160569	Aquatic invertebrates; other than crustaceans, molluscs, sea cucumbers, sea urchins, and jellyfish, prepared or preserved	3.321	FAO Assumed; average of all canned/preparations in containers derived CFs	Aquatic animals nei
160590	Molluscs and other aquatic invertebrates; prepared or preserved (excluding crustaceans)	3.489	FAO Assumed; average of all canned/preparations in containers derived CFs except crustaceans	Aquatic animals nei
169050	No Description	1	Base assumption due to lack of information	Marine fish nei

Supplementary Material Works Cited

(1) M. N. Santos, A. Garcia, Factors for conversion of fin weight into round weight for the blue shark (Prionace glauca). *Collect. Vol. Sci. Pap. ICCAT* **58**, 935–941 (2005).

(2) M. N. dos Santos, A. Garcia, New data on the ratio between fin and body weights for shark species caught by the Portuguese surface longline fleet. *Collect. Vol. Sci. Pap. ICCAT* **62**, 1592–1601 (2008).

(3) J. Mejuto, B. García-Cortés, J. Ortiz de Urbina, Ratios between the wet fin weight and body weights of blues shark (Prionace glauca) in the Spanish surface longline fleet during the period 1993-2006 and their impact on the ratio of sharks species combined. *Collect. Vol. Sci. Pap. ICCAT* **64**, 1492–1508 (2009).

(4) J. J. Waterman, "Measures, Stowage Rates and Yields of Fishery Products" (2001).
