# Supplementary material for: Disparities between sustainability of country-level seafood production and consumption
Source: PLoS One. 2024 Dec 2;19(12):e0313823. doi: 10.1371/journal.pone.0313823 (PMC11611205; doi:10.1371/journal.pone.0313823)
Supplement: S2 Table — The OHI gap filling analysis for estimating FMI values for countries not included in the original analysis by the Melnychuk group did not cover all possible reporters and partners in the GTA dataset. Where possible, we used the same model as OHI to continue gap filling (which relied on the Social Progress Index (SPI) and UN Georegions). In some cases SPI was not available, particularly for subregions of larger countries, groups of countries, or unrecognized territories. Below is a list of region/country equivalency and FMI calculation assumptions we made in order to complete the FMI data matching with the GTA data. (PDF) [file pone.0313823.s002.pdf]

Table S2. Reconciling FMI Gap Filling Data. The OHI gap filling analysis for estimating FMI values for countries not included in the original analysis by the Melnychuk group did not cover all possible reporters and partners in the GTA dataset. Where possible, we used the same model as OHI to continue gap filling (which relied on the Social Progress Index (SPI) and UN Georegions). In some cases SPI was not available, particularly for subregions of larger countries, groups of countries, or unrecognized territories. Below is a list of region/country equivalency and FMI calculation assumptions we made in order to complete the FMI data matching with the GTA data.

Country/Territory Lacking Data	Equivalent Country	Metric(s) Used to Equivocate	Method Used to Determine FMI	Reason excluded from OHI analysis
Hong Kong	China	FMI	Direct Equivalent	Chinese Territory
Macau	China	FMI	Direct Equivalent	Chinese Territory
US Virgin Islands	Puerto Rico	FMI	Direct Equivalent	US Territory
Martinique	Martinique and Guadaloupe	FMI	Direct Equivalent	Grouped with Guadaloupe
Serbia	Montenegro	FMI	Direct Equivalent	Landlocked
Austria	Austria	SPI/UN Georegion	OHI Gap Fill Equation	Landlocked
Belarus	Belarus	SPI/UN Georegion	OHI Gap Fill Equation	Landlocked
Botswana	Botswana	SPI/UN Georegion	OHI Gap Fill Equation	Landlocked
Czech Republic	Czech Republic	SPI/UN Georegion	OHI Gap Fill Equation	Landlocked
Hungary	Hungary	SPI/UN Georegion	OHI Gap Fill Equation	Landlocked
Kazakhstan	Kazakhstan	SPI/UN Georegion	OHI Gap Fill Equation	Landlocked
Luxembourg	Luxembourg	SPI/UN Georegion	OHI Gap Fill Equation	Landlocked
Slovakia	Slovakia	SPI/UN Georegion	OHI Gap Fill Equation	Landlocked
Switzerland	Switzerland	SPI/UN Georegion	OHI Gap Fill Equation	Landlocked
Bolivia	Bolivia	SPI/UN Georegion	OHI Gap Fill Equation	Landlocked
Paraguay	Paraguay	SPI/UN Georegion	OHI Gap Fill Equation	Landlocked
Kosovo	Montenegro	FMI	Direct Equivalent	Landlocked/ Unrecognized
Macedonia	Macedonia	SPI/UN Georegion	OHI Gap Fill Equation	Landlocked

Armenia	Armenia	SPI/UN Georegion	OHI Gap Fill Equation	Landlocked
Azerbaijan	Azerbaijan	SPI/UN Georegion	OHI Gap Fill Equation	NA
French Oceania	French Polynesia	FMI	Direct Equivalent	NA
French Terr. In Africa	Mayotte/ Reunion	FMI	mean(FMI of territories)	Territories
French Terr. In Central America	Guadeloupe/ St. Pierre	FMI	mean(FMI of territories)	Territories
Moldova	Moldova	SPI/UN Georegion	OHI Gap Fill Equation	Landlocked
Netherlands Antilles	Aruba/ Bonaire/ Curacao	FMI	mean(FMI of territories)	Grouped
Niger	Niger	SPI/UN Georegion	OHI Gap Fill Equation	Landlocked
Palestine	Israel	FMI	Direct Equivalent	Unrecognized
Spanish Africa, NES	Canary Islands	FMI	Direct Equivalent	Territories
Tajikistan	Tajikistan	SPI/UN Georegion	OHI Gap Fill Equation	Landlocked
Laos	Laos	SPI/UN Georegion	OHI Gap Fill Equation	Landlocked
Ships & Aircraft Stores	All from Original FMI/OHI Analysis	FMI	mean(FMI all countries)	Grouped
Liechtenstein	Switzerland	SPI/UN Georegion	OHI Gap Fill Equation	Landlocked
Vatican City State	Italy	FMI	Direct Equivalent	Landlocked
Turkmenistan	Turkmenistan	SPI/UN Georegion	OHI Gap Fill Equation	Landlocked
Unknown Countries	All from Original FMI/OHI Analysis	FMI	mean(FMI all countries)	Grouped
Burundi	Burundi	SPI/UN Georegion	OHI Gap Fill Equation	Landlocked
Chad	Chad	SPI/UN Georegion	OHI Gap Fill Equation	Landlocked
Ethiopia	Ethiopia	SPI/UN Georegion	OHI Gap Fill Equation	Landlocked
French Southern Terr.	St. Pierre/ Reunion	FMI	mean(FMI of territories)	Territories
Nepal	Nepal	SPI/UN Georegion	OHI Gap Fill Equation	Landlocked
Rwanda	Rwanda	SPI/UN Georegion	OHI Gap Fill Equation	Landlocked
Uganda	Uganda	SPI/UN Georegion	OHI Gap Fill Equation	Landlocked

Zambia	Zambia	SPI/UN Georegion	OHI Gap Fill Equation	Landlocked
Zimbabwe	Zimbabwe	SPI/UN Georegion	OHI Gap Fill Equation	Landlocked
Bhutan	Bhutan	SPI/UN Georegion	OHI Gap Fill Equation	Landlocked
Burkina Faso	Burkina Faso	SPI/UN Georegion	OHI Gap Fill Equation	Landlocked
St. Vincent & the Grenadines	Saint Vincent & the Grenadines	FMI	Direct Equivalent	Spelling Difference
U.S. Minor Outlying Is.	American Oceania	FMI	Direct Equivalent	Territories
British Terr. in Central Amer.	Anguilla/ Bermuda/ BVIs/ Caymans/ Montserrat/ Turks & Caicos	FMI	mean(FMI of territories)	Grouped
Central African Republic	Central African Republic	SPI/UN Georegion	OHI Gap Fill Equation	Landlocked
Unidentified Country	All from Original FMI/OHI Analysis	FMI	mean(FMI all countries)	Grouped
Comoros	Comoros	SPI/UN Georegion	OHI Gap Fill Equation	Landlocked
Kyrgyzstan	Kyrgyzstan	SPI/UN Georegion	OHI Gap Fill Equation	Landlocked
Mali	Mali	SPI/UN Georegion	OHI Gap Fill Equation	Landlocked
Mongolia	Mongolia	SPI/UN Georegion	OHI Gap Fill Equation	Landlocked
Other Oceania, N.E.S.	Oceania	FMI	mean(FMI of Oceania groups)	Grouped
South Sudan	South Sudan	SPI/UN Georegion	OHI Gap Fill Equation	Landlocked
Duty Free (Cartagena)	Colombia	FMI	Direct Equivalent	NA
Duty Free	All from Original FMI/OHI Analysis	FMI	mean(FMI all countries)	Grouped
Other Countries, NES	All from Original FMI/OHI Analysis	FMI	mean(FMI all countries)	Grouped
Not Determin Extra EU Trade	EU	FMI	mean(FMI of non-EU countries)	Grouped
Not Determin Intra EU Trade	EU	FMI	mean(FMI of EU countries)	Grouped

Afghanistan	Afghanistan	SPI/UN Georegion	OHI Gap Fill Equation	Landlocked
San Marino	Italy	FMI	Direct Equivalent	Landlocked
International Waters	All from Original FMI/OHI Analysis	FMI	mean(FMI all countries)	Grouped
Not Determined	All from Original FMI/OHI Analysis	FMI	mean(FMI all countries)	Grouped
Uzbekistan	Uzbekistan	SPI/UN Georegion	OHI Gap Fill Equation	Landlocked
Andorra	Switzerland	SPI/UN Georegion	OHI Gap Fill Equation	Landlocked
Wallis & Futuna	Wallis and Futuna Islands	FMI	Direct Equivalent	Spelling Difference
Extra EU Stores & Provisions	EU	FMI	mean(FMI of EU countries)	Grouped
Swaziland	Swaziland	SPI/UN Georegion	OHI Gap Fill Equation	Landlocked
Czechoslovakia (Former)	Czechoslovakia (Former)	SPI/UN Georegion	OHI Gap Fill Equation	Landlocked
Stores & Provisions	All from Original FMI/OHI Analysis	FMI	mean(FMI all countries)	Grouped
Malawi	Malawi	SPI/UN Georegion	OHI Gap Fill Equation	Landlocked
Yugoslavia	Bosnia & Herzegovina/ Croatia/ Serbia/ Slovenia	FMI	mean(FMI of countries)	Unrecognized
Non-EU Suppression	All non-European Countries	FMI	mean(FMI of countries)	Grouped
Other Asia, N.E.S.	Asia	FMI	mean(FMI of Asia groups)	Grouped
Pacific Is. (Trust Terr.)	Marshall Islands/ Micronesia/ Palau/ Northern Mariana Islands	FMI	mean(FMI of territories)	Unrecognized
Antarctica	Argentina/ Australia/ Chile/ France/ New Zealand/ Norway/	FMI	mean(FMI of countries with territorial claims on Antarctica)	Unrecognized

	UK			
Intra EU Stores & Provisions	EU	FMI	mean(FMI of EU countries)	Grouped
Nieu	Niue	FMI	Direct Equivalent	Spelling Difference
Storage Deposits	All from Original FMI/OHI Analysis	FMI	mean(FMI all countries)	Grouped
British Indian Ocean Terr.	Seychelles	FMI	Direct Equivalent	Territory/ Grouped
Lesotho	Lesotho	SPI/UN Georegion	OHI Gap Fill Equation	Landlocked
Midway Islands	American Samoa/ Guam/ Northern Mariana Islands	FMI	mean(FMI of other American Oceania territories)	Territory
FTZ-Aegean	Turkey	FMI	Direct Equivalent	Territory
FTZ-Bursa	Turkey	FMI	Direct Equivalent	Territory
FTZ-Menemen Leather	Turkey	FMI	Direct Equivalent	Territory
West Bank	Israel	FMI	Direct Equivalent	Unrecognized
Commercial or Military Secret	All from Original FMI/OHI Analysis	FMI	mean(FMI all countries)	Grouped
FTZ-Colon	Panama	FMI	Direct Equivalent	Territory
FTZ-Ecuador	Ecuador	FMI	Direct Equivalent	Territory
Other North America, N.E.S.	North America	FMI	mean(FMI of North America Groups)	Grouped
Austral. Oceania	Austral. Oceania	FMI	mean(FMI of Austral. Oceania Groups)	Grouped
Central/South Amer., NES	Central/South America	FMI	mean(FMI of Central/South America Groups)	Grouped
Oth. Africa, N.E.S.	Africa	FMI	mean(FMI of Africa Groups)	Grouped
Oth. West Europe	West Europe	FMI	mean(FMI of West Europe Groups)	Grouped
Other Latin America, N.E.S.	Latin America	FMI	mean(FMI of Latin America Groups)	Grouped
Tahiti	French Polynesia	FMI	Direct Equivalent	Territory
Products Outside Ter. Waters	All from Original FMI/OHI Analysis	FMI	mean(FMI all countries)	Grouped
Returned Bahrain Goods	Bahrain	FMI	Direct Equivalent	NA

S. Georgia & S. Sandwich	Falkland Islands	FMI	Direct Equivalent	Territory
BES Islands	Bonaire/ Sint Eustatius/ Saba	FMI	mean(FMI of territories)	Grouped/ Territories
St. Barthelemy	French Terr. In Central America	FMI	Direct Equivalent	Territory
Heard & McDonald Is.	Austral. Oceania	FMI	mean(FMI of Austral. Oceania Groups)	Grouped
Products Within Ter. Waters	All from Original FMI/OHI Analysis	FMI	mean(FMI all countries)	Grouped
European Union	EU	FMI	mean(FMI of EU countries)	Grouped
Other South American Countries	South America	FMI	mean(FMI of South America Groups)	Grouped
St. Brandon & Outer Islands	Mauritius	FMI	Direct Equivalent	Territory
Svalbard & Jan Mayen	Norway	FMI	Direct Equivalent	Territory
British Terr. in Africa	Ghana/ Kenya/ Nigeria/ Sierra Leone/ South Africa/ Tanzania	FMI	Mean(FMI of African Commonwealth Countries in original OHI analysis)	Grouped
High Seas	All from Original FMI/OHI Analysis	FMI	mean(FMI all countries)	Grouped
Aland Islands	Finland	FMI	Direct Equivalent	Territory
FTZ-Mersin	Turkey	FMI	Direct Equivalent	Territory
FTZ-Jebel Ali	UAE	FMI	Direct Equivalent	Territory
Bouvet Island	Norway	FMI	Direct Equivalent	Territory
Other Middle & Near Eastern	West Asia	FMI	mean(FMI of West Asia Countries)	Grouped
EU Suppression	EU	FMI	mean(FMI of EU countries)	Grouped
Other Australian Terr.	Christmas Island/ Cocos Islands/ Norfolk Island	FMI	mean(FMI of territories)	Grouped
British Terr. in Oceania	Pitcairn Islands	FMI	Direct Equivalent	Territory
Dutch Terr. in America	Netherlands Antilles	FMI	Direct Equivalent	Territories/ Grouped

Rajaee Port Special Econ. Zone	Iran	FMI	Direct Equivalent	Territory
Free Trade Zones	All from Original FMI/OHI Analysis	FMI	mean(FMI all countries)	Grouped
American Oceania	American Samoa/ Guam/ Northern Mariana Islands	FMI	mean(FMI of territories)	Grouped
FTZ-Chabahar	Iran	FMI	Direct Equivalent	Territory
Other Europe, N.E.S.	Europe	FMI	mean(FMI of Europe countries)	Grouped
