# Supplementary material for: Disparities between sustainability of country-level seafood production and consumption
Source: PLoS One. 2024 Dec 2;19(12):e0313823. doi: 10.1371/journal.pone.0313823 (PMC11611205; doi:10.1371/journal.pone.0313823)
Supplement: S3 Table — Some trade partners did not have an associated proportion of production. This largely happened in instances where the trade partners did not align with the FAO countries (e.g. grouped EU trade relationships). For those instances, we decided the production proportion on a case-by-case basis, usually taking the mean for countries included in the group. Below is a list of all such trade partners, whether the product in question was a fish or invertebrate, and how we derived the estimate of production proportion. (PDF) [file pone.0313823.s003.pdf]

Table S3. Proportion of Production Estimates for Trade Partners. Some trade partners did not have an associated proportion of production. This largely happened in instances where the trade partners did not align with the FAO countries (e.g. grouped EU trade relationships). For those instances, we decided the production proportion on a case-by-case basis, usually taking the mean for countries included in the group. Below is a list of all such trade partners, whether the product in question was a fish or invertebrate, and how we derived the estimate of production proportion.

Trade Partner	Coarse Group	Derivation
Kosovo	Fish	mean(Pp) for Montenegro fish
North Korea	Invert	median(Pp) invert
North Korea	Fish	median(Pp) fish
Netherlands Antilles	Fish	mean(Pp) for fish Aruba, Curacao
Liechtenstein	Invert	mean(Pp) for inverts Switzerland
Andorra	Fish	mean(Pp) for fish Switzerland
European Union	Fish	mean(Pp) for fish EU
European Union	Invert	mean(Pp) for invert EU
FTZ-Jebel Ali	Fish	mean(Pp) for fish UAE
Liechtenstein	Fish	mean(Pp) for fish Switzerland
Returned Bahrain Goods	Invert	mean(Pp) for invert Bahrain
Returned Bahrain Goods	Fish	mean(Pp) for fish Bahrain
Not Determin Extra EU Trade	Fish	mean(Pp) for fish EU
Not Determin Extra EU Trade	Invert	mean(Pp) for invert EU
Unknown Countries	Invert	median(Pp) invert
Gibraltar	Invert	mean(Pp) for invert Spain
Gibraltar	Fish	mean(Pp) for fish Spain
Unknown Countries	Fish	median(Pp) fish
Not Determined	Invert	median(Pp) invert
Unidentified Country	Fish	median(Pp) fish
Not Determin Intra EU Trade	Invert	mean(Pp) for invert EU
U.S. Minor Outlying Is.	Fish	mean(Pp) for fish American Samoa, Northern Mariana Islands & Guam
U.S. Minor Outlying Is.	Invert	mean(Pp) for invert American Samoa, Northern Mariana Islands & Guam
Commercial or Military Secret	Invert	median(Pp) invert

Commercial or Military Secret	Fish	median(Pp) fish
French Oceania	Fish	mean(Pp) for fish French Polynesia
Afghanistan	Invert	FAO has no record of invert production in Afghanistan
Other Latin America, N.E.S.	Invert	mean(Pp) for invert Latin America
Duty Free (Cartagena)	Fish	mean(Pp) for fish Colombia
Bouvet Island	Invert	mean(Pp) for invert Norway
FTZ-Colon	Fish	mean(Pp) for fish Ecuador
Duty Free	Fish	median(Pp) fish
Duty Free	Invert	median(Pp) invert
Other Countries, NES	Invert	median(Pp) invert
Other Countries, NES	Fish	median(Pp) fish
Not Determin Intra EU Trade	Fish	mean(Pp) for fish EU
Christmas Island	Fish	mean(Pp) for fish Austral. Oceania
FTZ-Ecuador	Invert	mean(Pp) for invert Ecuador
FTZ-Ecuador	Fish	mean(Pp) for fish Ecuador
International Waters	Fish	median(Pp) fish
Not Determined	Fish	median(Pp) fish
Other North America, N.E.S.	Invert	mean(Pp) for invert US, Canada, Mexico
Other North America, N.E.S.	Fish	mean(Pp) for fish US, Canada, Mexico
Other South American Countries	Fish	mean(Pp) for fish South America
Andorra	Invert	mean(Pp) for inverts Switzerland
Austral. Oceania	Invert	mean(Pp) for invert Austral. Oceania
Austral. Oceania	Fish	mean(Pp) for fish Austral. Oceania
Central/South Amer., NES	Invert	mean(Pp) for invert Latin America
Central/South Amer., NES	Fish	mean(Pp) for fish Latin America
Oth. Africa, N.E.S.	Invert	mean(Pp) for invert Africa
Oth. Africa, N.E.S.	Fish	mean(Pp) for fish Africa
Oth. West Europe	Fish	mean(Pp) for fish West Europe
Oth. West Europe	Invert	mean(Pp) for invert West Europe
Other Asia, N.E.S.	Fish	mean(Pp) for fish Asia
Other Europe, N.E.S.	Fish	mean(Pp) for fish Europe
Other Middle & Near Eastern	Invert	mean(Pp) for invert West Asia
Pitcairn	Invert	FAO has no record of invert production in Pitcairn
Tokelau	Invert	FAO has no record of invert production in Tokelau
Vatican City State	Invert	Assumed no production from Vatican
Vatican City State	Fish	Assumed no production from Vatican
Unidentified Country	Invert	median(Pp) invert
FTZ-Chabahar	Fish	mean(Pp) for fish Turkey

Canary Islands	Invert	mean(Pp) for invert Spain
Other Australian Terr.	Fish	mean(Pp) for fish Austral. Oceania
Western Sahara	Invert	mean(Pp) for invert Morocco
Intra EU Stores & Provisions	Fish	mean(Pp) for fish EU
St. Brandon & Outer Islands	Fish	mean(Pp) for fish Mauritius
Monaco	Invert	FAO has no record of invert production in Monaco
Svalbard & Jan Mayen	Invert	mean(Pp) for invert in Norway
EU Suppression	Invert	mean(Pp) for invert EU
EU Suppression	Fish	mean(Pp) for fish EU
High Seas	Fish	median(Pp) fish
Non-EU Suppression	Invert	mean(Pp) for invert not Europe
Non-EU Suppression	Fish	mean(Pp) for fish not Europe
Antarctica	Fish	mean(Pp) for fish Antarctica claim
Free Trade Zones	Invert	median(Pp) invert
Free Trade Zones	Fish	median(Pp) fish
FTZ-Colon	Invert	mean(Pp) for invert Ecuador
Storage Deposits	Fish	median(Pp) fish
Storage Deposits	Invert	median(Pp) invert
Antarctica	Invert	mean(Pp) for invert Antarctica claim
International Waters	Invert	median(Pp) invert
San Marino	Fish	mean(Pp) for fish Italy
Nauru	Invert	FAO has no record of invert production in Nauru
Other Latin America, N.E.S.	Fish	mean(Pp) for fish Latin America
Midway Islands	Fish	mean(Pp) for fish American Samoa, Northern Mariana Islands & Guam
Svalbard & Jan Mayen	Fish	mean(Pp) for fish Norway
Products Outside Ter. Waters	Fish	median(Pp) fish
Products Outside Ter. Waters	Invert	median(Pp) invert
Cocos Islands	Fish	mean(Pp) for fish Austral. Oceania
Norfolk Island	Fish	mean(Pp) for fish Austral. Oceania
