# Supplementary material for: Disparities between sustainability of country-level seafood production and consumption
Source: PLoS One. 2024 Dec 2;19(12):e0313823. doi: 10.1371/journal.pone.0313823 (PMC11611205; doi:10.1371/journal.pone.0313823)
Supplement: S4 Table — Production sustainability is the FMIP for each country, and mean consumption sustainability is the mean of the three FMIC derivations. We calculated the percent change between these values for each country by subtracting the mean consumption sustainability from the production sustainability and dividing by the production sustainability and multiplying by 100 ((FMIP−mean(FMIC)/FMIP)*100). The nature of this equation means that positive percent change values occur for countries that have a decrease in consumption sustainability compared to production sustainability and negative values occur for countries that have an increase. Major differences compared to core analysis: Notably, the top 25 seafood producing countries changed with the removal of potential aquaculture products. The top 6 countries remained the same. New Zealand and South Africa made the top 25, while Argentina and India are no longer on the list. (PDF) [file pone.0313823.s004.pdf]

Table S4. Percent change in production and consumption sustainability for the top 25 seafood producing countries using the GTA wild capture data (according to FAO data). Production sustainability is the FMI_P for each country, and mean consumption sustainability is the mean of the three FMI_C derivations. We calculated the percent change between these values for each country by subtracting the mean consumption sustainability from the production sustainability and dividing by the production sustainability and multiplying by 100 $((FMI_P - mean(FMI_C)/FMI_P)*100)$. The nature of this equation means that positive percent change values occur for countries that have a decrease in consumption sustainability compared to production sustainability and negative values occur for countries that have an increase. *Major differences compared to core analysis*: Notably, the top 25 seafood producing countries changed with the removal of potential aquaculture products. The top 6 countries remained the same. New Zealand and South Africa made the top 25, while Argentina and India are no longer on the list.

Country	Production Sustainability (FMI_P)	Mean Consumption Sustainability mean(FMI_C)	Percent Change (%)
United States of America	0.932	0.739	23.43
Iceland	0.929	0.862	7.51
Norway	0.865	0.803	7.59
Denmark	0.842	0.792	6.09
Russia	0.838	0.754	10.63
Canada	0.834	0.740	12.02
New Zealand	0.785	0.714	14.48
South Africa	0.787	0.736	6.70
United Kingdom	0.785	0.707	10.54
Chile	0.706	0.641	9.87
South Korea	0.692	0.671	3.09
Spain	0.690	0.648	6.35

Japan	0.679	0.638	6.26
France	0.663	0.631	5.03
Morocco	0.622	0.617	0.87
Peru	0.613	0.610	0.47
Taiwan	0.609	0.583	4.39
Mexico	0.588	0.565	3.91
Indonesia	0.458	0.502	-8.79
Turkey	0.431	0.518	-17.64
Malaysia	0.386	0.422	-8.74
Philippines	0.381	0.425	-10.56
China	0.370	0.429	-14.32
Thailand	0.313	0.393	-22.401
Brazil	0.251	0.385	-40.88
