# Supplementary material for: Disparities between sustainability of country-level seafood production and consumption
Source: PLoS One. 2024 Dec 2;19(12):e0313823. doi: 10.1371/journal.pone.0313823 (PMC11611205; doi:10.1371/journal.pone.0313823)
Supplement: S5 Table — SE: Standard Error. (PDF) [file pone.0313823.s005.pdf]

Table S5. Linear model results estimating the relationship between FMI_P and the three FMI_C derivations using the GTA wild capture data (Model Equation: $FMI_C \sim FMI_P$). SE: Standard Error.

FMI_C Derivation	Intercept estimate	Intercept SE	Slope estimate	Slope SE	Slope t value	p-value	R^2
Proportional	0.202	0.024	0.612	0.035	17.272	<0.0001	0.81
Guillen	0.112	0.008	0.798	0.011	69.75	<0.0001	0.99
Gephart	0.285	0.020	0.513	0.030	17.55	<0.0001	0.81
