# Supplementary material for: Disparities between sustainability of country-level seafood production and consumption
Source: PLoS One. 2024 Dec 2;19(12):e0313823. doi: 10.1371/journal.pone.0313823 (PMC11611205; doi:10.1371/journal.pone.0313823)
Supplement: S1 Fig — Each point represents a single country with the line showing the hypothetical one-to-one relationship that would occur if the datasets matched. (PDF) [file pone.0313823.s006.pdf]

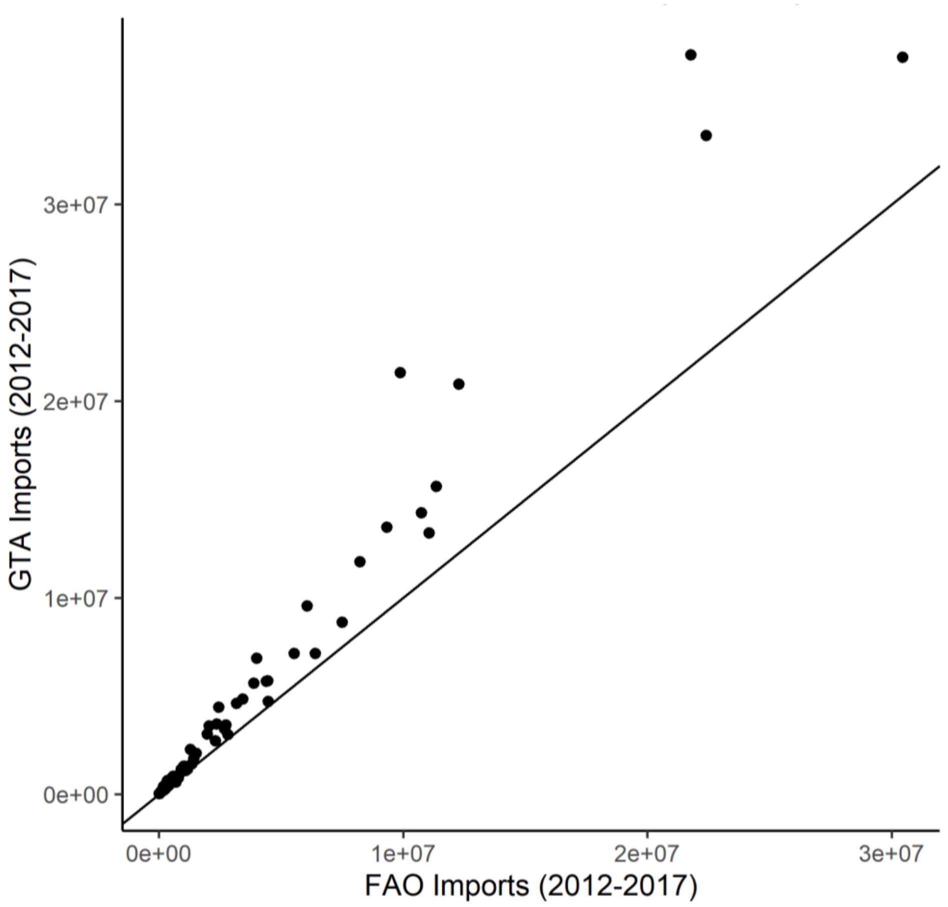

Fig S1. Comparison of import quantities by country from the GTA and FAO datasets. Each point represents a single country with the line showing the hypothetical one-to-one relationship that would occur if the datasets matched.
