# Supplementary material for: Disparities between sustainability of country-level seafood production and consumption
Source: PLoS One. 2024 Dec 2;19(12):e0313823. doi: 10.1371/journal.pone.0313823 (PMC11611205; doi:10.1371/journal.pone.0313823)
Supplement: S2 Fig — (A) Depiction of the difference between production and consumption sustainability as measured by the Fisheries Management Index (FMI). The colored bars show the difference between the production sustainability (FMIP—squares) and the mean of the consumption sustainability derivations (FMIC—triangles), with red indicating a decrease in consumption sustainability compared to production sustainability and green indicating an increase. The gray lines show the range, and the asterisks show the direct estimates of each of the consumption sustainability derivations (FMIC−Proportional, Guillen, and Gephart). Countries that produce more sustainable seafood than they are consuming occur above the dotted line, while countries that produce less sustainable seafood than they are consuming occur below. (B) The second panel displays the relative proportion of exports (gray bar) compared to combined total imports (dark blue bar) and production (light blue bar) for each country. Note that we do not know what proportion of exports is made up of production versus imports, and thereby do not know what proportions of production and imports are left for consumption. This accounts for the uncertainty in the consumption sustainability estimates in the left panel. (PDF) [file pone.0313823.s007.pdf]

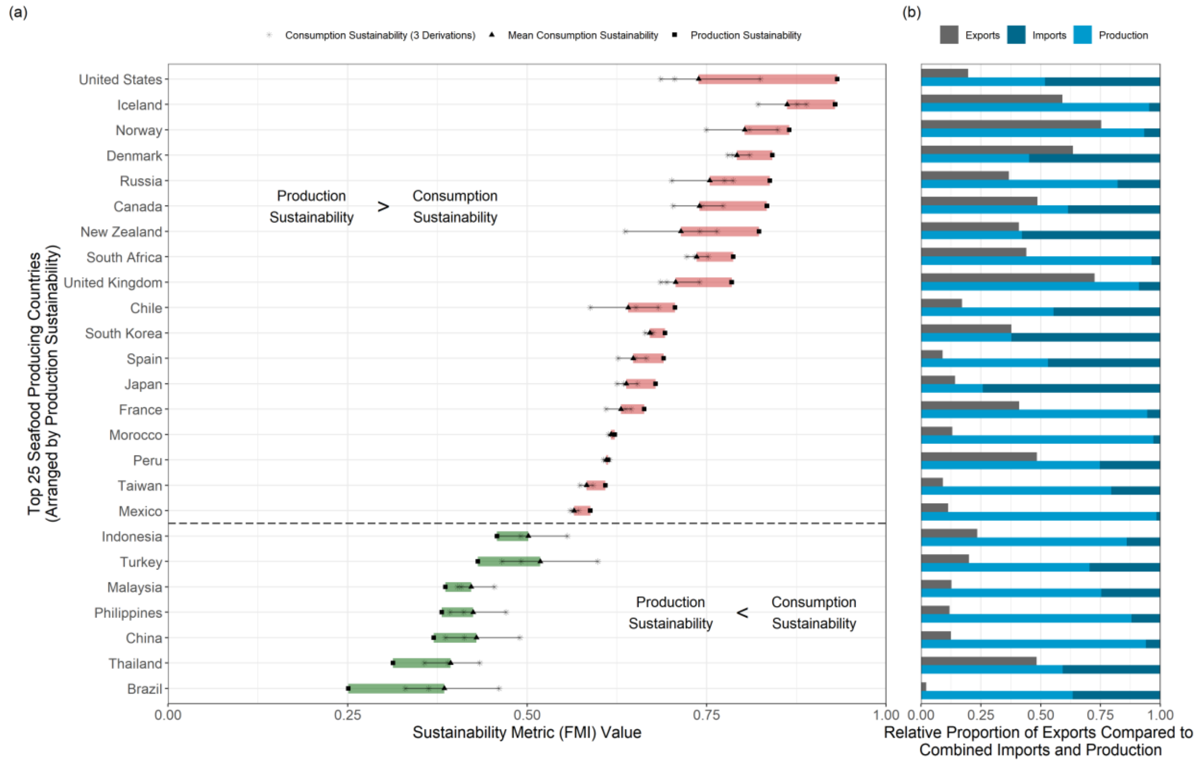

Fig S2. Aquaculture Exclusion Analysis: Plot showing disparity in production and consumption sustainability and the relative proportions of imports, exports, and production for the top 25 seafood producing countries from 2012-2017. (A) Depiction of the difference between production and consumption sustainability as measured by the Fisheries Management Index (FMI). The colored bars show the difference between the production sustainability (FMI_P - squares) and the mean of the consumption sustainability derivations (FMI_C - triangles), with red indicating a decrease in consumption sustainability compared to production sustainability and green indicating an increase. The gray lines show the range, and the asterisks show the direct estimates of each of the consumption sustainability derivations (FMI_C – Proportional, Guillen, and Gephart). Countries that produce more sustainable seafood than they are consuming occur above the dotted line, while countries that produce less sustainable seafood than they are consuming occur below. (B) The second panel displays the relative proportion of exports (gray bar) compared to combined total imports (dark blue bar) and production (light blue bar) for each country. Note that we do not know what proportion of exports is made up of production versus imports, and thereby do not know what proportions of production and imports are left for consumption. This accounts for the uncertainty in the consumption sustainability estimates in the left panel.
