# Supplementary material for: Disparities between sustainability of country-level seafood production and consumption
Source: PLoS One. 2024 Dec 2;19(12):e0313823. doi: 10.1371/journal.pone.0313823 (PMC11611205; doi:10.1371/journal.pone.0313823)
Supplement: S3 Fig — The points depict the raw data (countries), while the lines show the linear model results. The shaded areas around the lines denote the 95% confidence intervals for the linear model fit. The different colors denote the different consumption sustainability (FMIC) derivations. The black line shows the hypothetical direct 1:1 relationship between the two variables for comparison. (PDF) [file pone.0313823.s008.pdf]

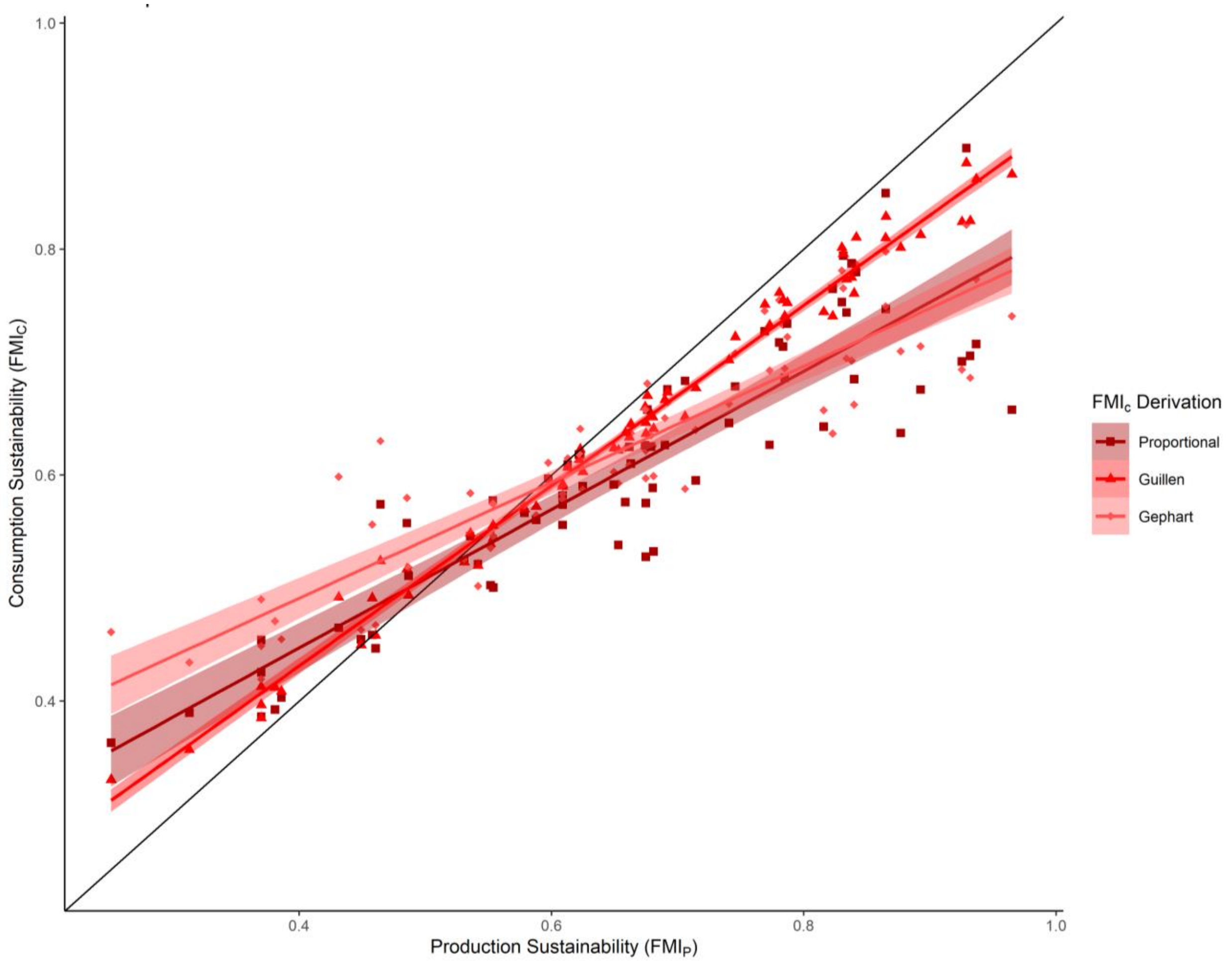

Fig S3. Aquaculture Exclusion Analysis: Plot comparing production to consumption sustainability across all countries analyzed. The points depict the raw data (countries), while the lines show the linear model results. The shaded areas around the lines denote the 95% confidence intervals for the linear model fit. The different colors denote the different consumption sustainability (FMI_C) derivations. The black line shows the hypothetical direct 1:1 relationship between the two variables for comparison.
