# Supplementary material for: Disparities between sustainability of country-level seafood production and consumption
Source: PLoS One. 2024 Dec 2;19(12):e0313823. doi: 10.1371/journal.pone.0313823 (PMC11611205; doi:10.1371/journal.pone.0313823)
Supplement: S4 Fig — Aquaculture exclusion analysis: The proportion of global exports (a) and imports (b) from 2012–2017 for each country analyzed arranged by FMIP. The size and color of the points shows the number of trade partners for each country (note difference in scale of y-axis). The top five exporters and importers are labeled in each panel. (PDF) [file pone.0313823.s009.pdf]

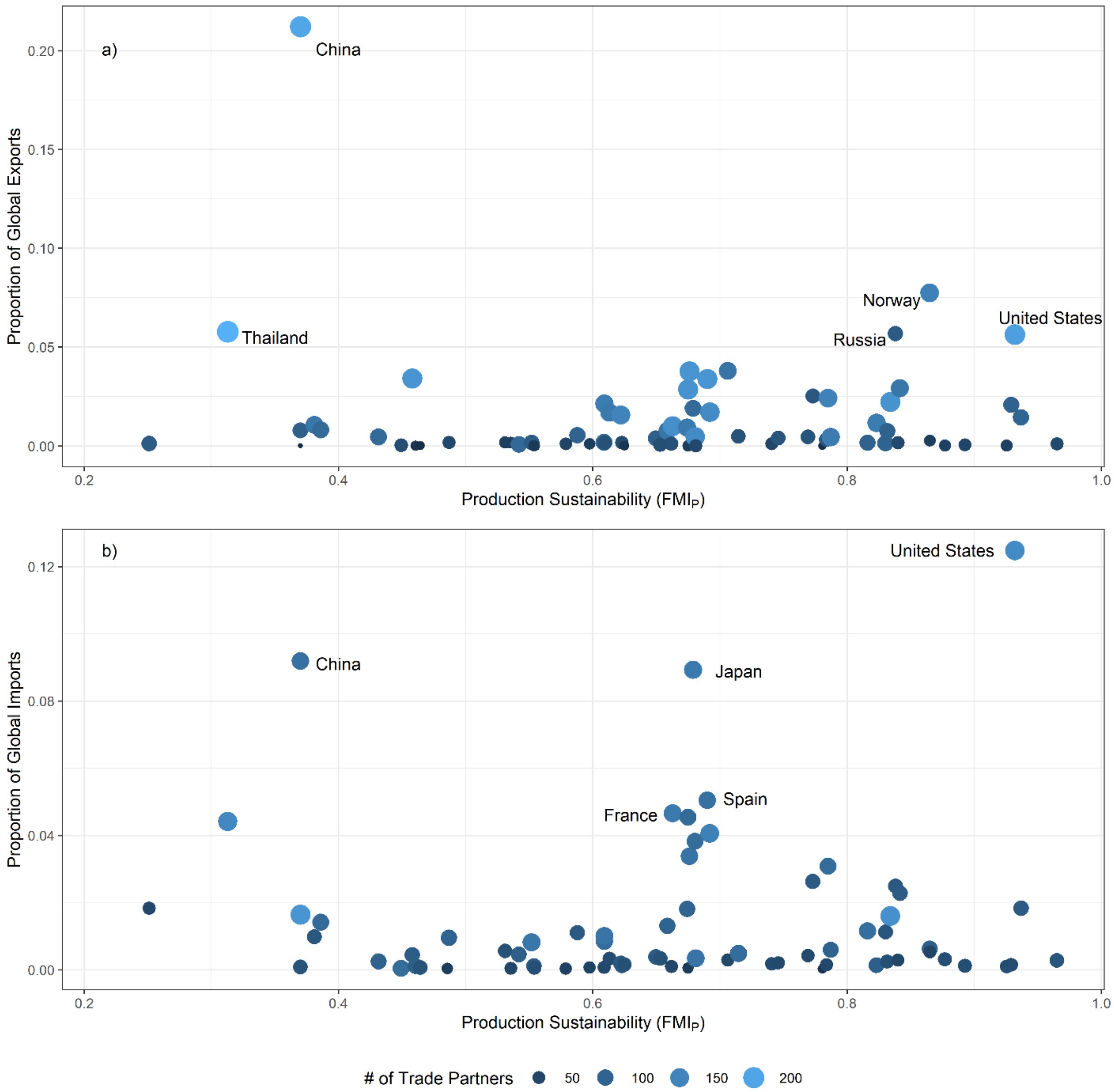

Fig S4. Aquaculture Exclusion Analysis: The proportion of global exports (a) and imports (b) from 2012-2017 for each country analyzed arranged by FMI$_P$. The size and color of the points shows the number of trade partners for each country (note difference in scale of y-axis). The top five exporters and importers are labeled in each panel.
