# Supplementary material for: Disparities between sustainability of country-level seafood production and consumption
Source: PLoS One. 2024 Dec 2;19(12):e0313823. doi: 10.1371/journal.pone.0313823 (PMC11611205; doi:10.1371/journal.pone.0313823)
Supplement: S5 Fig — Aquaculture exclusion analysis: Figure depicting (a) imports to the United States of America (USA) from their top 25 import partners and (b) exports from China to their top 25 export partners from 2012–2017. In the maps, China and the USA are shaded based on their own production sustainability. The lines and points show the origin of imports to the USA (a) and destination of exports from China (b), respectively, with the color denoting the production sustainability of the trade partner. The thickness and darkness of the lines and points are scaled to the magnitude of the trade going to/from each country. The bar plots depict the proportion of total USA imports or Chinese exports attributable to each of top 25 trade partners, respectively. The USA’s top 25 import partners account for 94.34% of total imports to the USA from 2012–2017. China’s top 25 export partners account for 89.90% of total exports from China from 2012–2017. The bars are shaded based on the trade partner’s production sustainability. (PDF) [file pone.0313823.s010.pdf]

Blincow et al. PLoSONE Submission - Supplementary Material

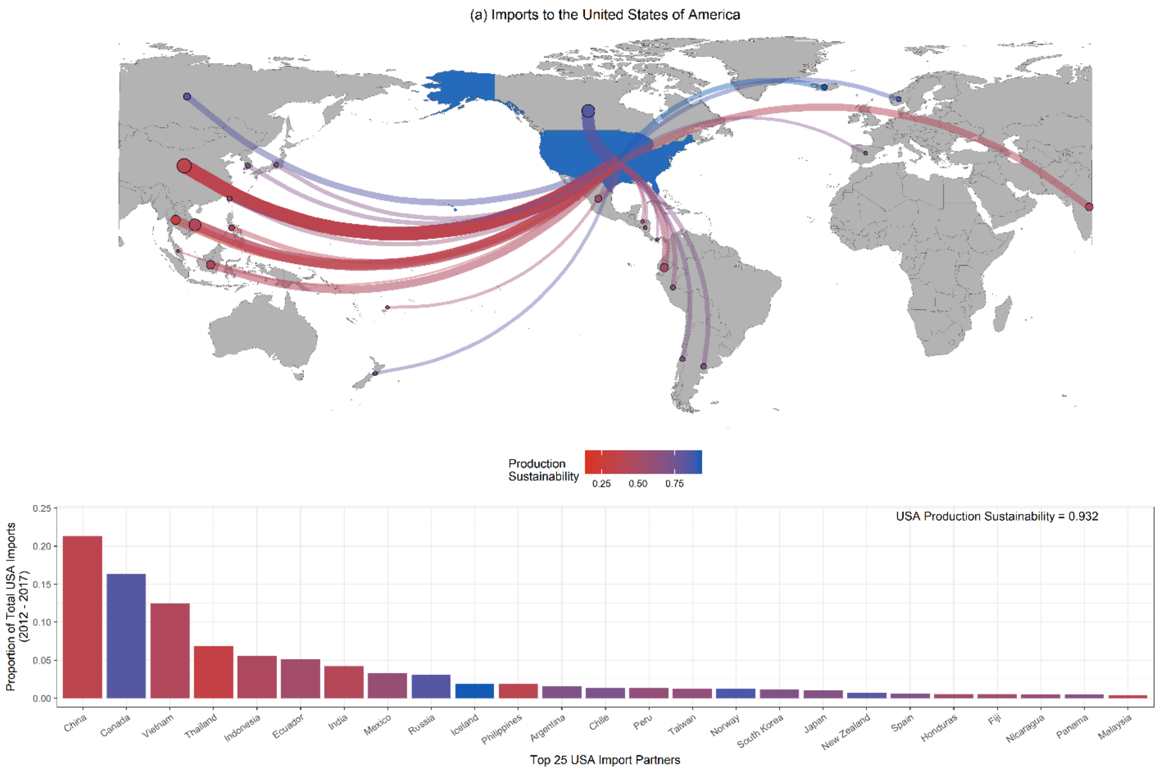

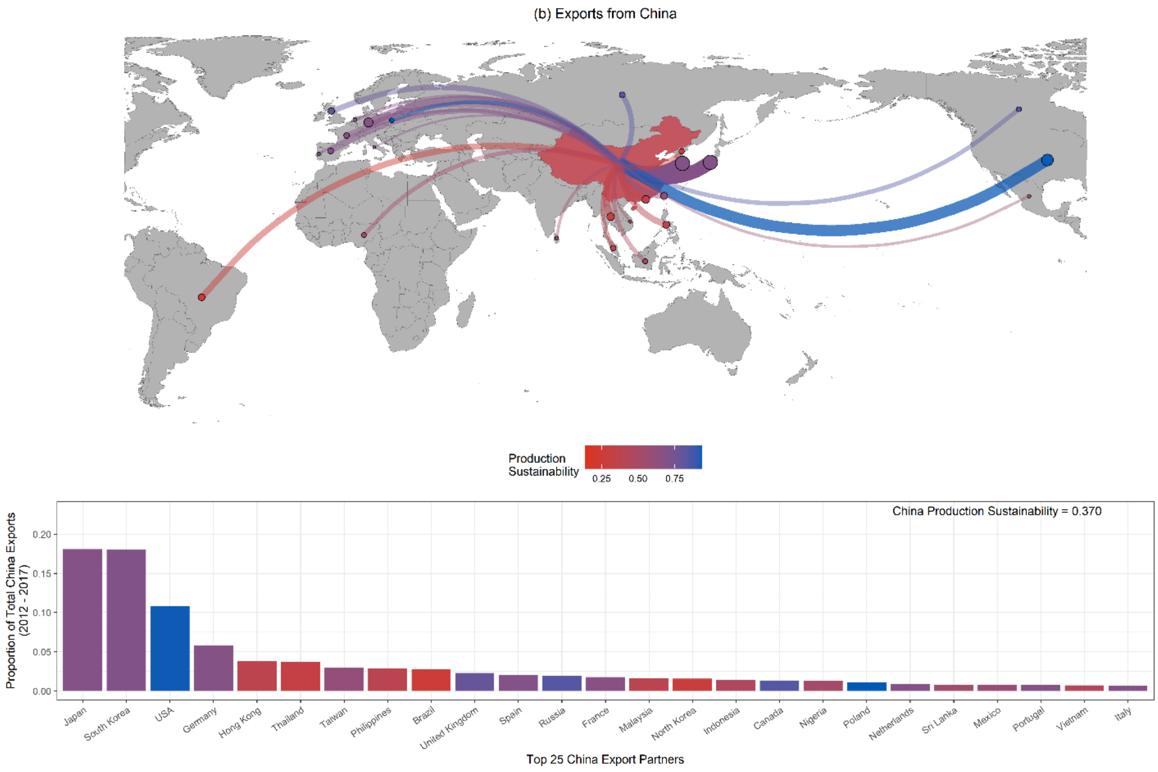

Fig S5. Aquaculture Exclusion Analysis: Figure depicting (a) imports *to* the United States of America (USA) from their top 25 import partners and (b) exports *from* China to their top 25 export partners from 2012-2017. In the maps, China and the USA are shaded based on their own production sustainability. The lines and points show the origin of imports to the USA (a) and destination of exports from China (b), respectively, with the color denoting the production sustainability of the trade partner. The thickness and darkness of the lines and points are scaled to the magnitude of the trade going to/from each country. The bar plots depict the proportion of total USA imports or Chinese exports attributable to each of top 25 trade partners, respectively. The USA's top 25 import partners account for 94.34% of total imports to the USA from 2012-2017. China's top 25 export partners account for 89.90% of total exports from China from 2012-2017. The bars are shaded based on the trade partner's production sustainability. The base maps were made using public domain data from Natural Earth (naturalearthdata.com).
